# Effects of dietary intervention on human diseases: molecular mechanisms and therapeutic potential

**DOI:** 10.1038/s41392-024-01771-x

**Published:** 2024-03-11

**Authors:** Yu-Ling Xiao, Yue Gong, Ying-Jia Qi, Zhi-Ming Shao, Yi-Zhou Jiang

**Affiliations:** 1 Key Laboratory of Breast Cancer in Shanghai, Department of Breast Surgery, Fudan University Shanghai Cancer Center, Shanghai, China; 2 Department of Oncology, Shanghai Medical College, Fudan University, Shanghai, 200032 China

**Keywords:** Cancer metabolism, Immunotherapy, Cancer imaging

## Abstract

Diet, serving as a vital source of nutrients, exerts a profound influence on human health and disease progression. Recently, dietary interventions have emerged as promising adjunctive treatment strategies not only for cancer but also for neurodegenerative diseases, autoimmune diseases, cardiovascular diseases, and metabolic disorders. These interventions have demonstrated substantial potential in modulating metabolism, disease trajectory, and therapeutic responses. Metabolic reprogramming is a hallmark of malignant progression, and a deeper understanding of this phenomenon in tumors and its effects on immune regulation is a significant challenge that impedes cancer eradication. Dietary intake, as a key environmental factor, can influence tumor metabolism. Emerging evidence indicates that dietary interventions might affect the nutrient availability in tumors, thereby increasing the efficacy of cancer treatments. However, the intricate interplay between dietary interventions and the pathogenesis of cancer and other diseases is complex. Despite encouraging results, the mechanisms underlying diet-based therapeutic strategies remain largely unexplored, often resulting in underutilization in disease management. In this review, we aim to illuminate the potential effects of various dietary interventions, including calorie restriction, fasting-mimicking diet, ketogenic diet, protein restriction diet, high-salt diet, high-fat diet, and high-fiber diet, on cancer and the aforementioned diseases. We explore the multifaceted impacts of these dietary interventions, encompassing their immunomodulatory effects, other biological impacts, and underlying molecular mechanisms. This review offers valuable insights into the potential application of these dietary interventions as adjunctive therapies in disease management.

## Introduction

Nutrients play a crucial role in regulating various physiological processes.^1^ The main source of nutrients is usually considered to be diet. The quantity, quality, and composition of the food consumed, as well as the timing of meals, directly impact human health by influencing the availability of nutrients.^2^ Although there have been advancements in understanding the link between diet and disease in recent years, there is still much to learn about how specific dietary components affect disease risk and prevention.^3^

Epidemiological studies have linked various dietary patterns to cancer and other diseases.^4^ For instance, diets high in saturated fats and sugars have been associated with an increased risk of cardiovascular diseases (CVD) and type 2 diabetes.^5^ Conversely, diets rich in fiber, fruits, and vegetables are associated with a lower risk of these conditions.^6^ Similarly, conditions such as osteoporosis and certain neurological disorders have also shown links to dietary patterns, highlighting the broad influence of diet on overall health.^7,8^ In the context of cancer, increased consumption of alcohol and red or processed meat is associated with a heightened risk of cancer, whereas adherence to a Mediterranean dietary pattern—characterized by high intake of fruits, vegetables, whole grains, legumes, fish, and olive oil, along with moderate consumption of dairy products such as yogurt—may confer protective effects against carcinogenesis.^9,10^ Similarly, a strong adherence to the plant-based Paleolithic diet and a Paleolithic-like lifestyle has been found to significantly reduce the risk of colorectal cancer (CRC), especially in individuals with a body mass index (BMI) less than 30.^11^ Although many cancer patients are interested in using dietary intervention to improve cancer therapy outcomes or even using it as a key component of the therapeutic process,^12^ there is currently no solid evidence showing that any nutrition-related regimen can be a primary treatment for cancer.^13^ However, preclinical studies suggest that calorie and energy restrictions can hinder tumor growth and progression and increase the efficacy of chemotherapy and radiotherapy.^14,15^ A rising number of clinical trials are exploring the impact of dietary interventions or nutritional supplements in conjunction with standard antitumor therapies, with some showing clinical benefits.^16,17^

Diet is a crucial source of nutrients for tumors and has emerged as a key component in determining whole-body metabolism.^18^ The nutrients in the tumor microenvironment (TME) largely regulate tumor cell and immune cell metabolism.^19^ Recent evidence suggests that metabolic reprogramming, a crucial hallmark of cancer, involves several metabolic adaptations by tumor cells to sustain proliferation and metastasis in the TME.^19–21^ The TME constitutes a multifaceted and dynamic ecosystem comprising an assortment of cell types, including tumor cells, immune cells, and stromal cells, in addition to components of the extracellular matrix. The interplay among these constituents, along with the challenging environmental conditions, exerts a significant influence on the growth trajectory and progression of tumors.^22^ For example, oxygen levels within the TME can vary due to increased metabolic demand from rapidly proliferating tumor cells, resulting in low oxygen tension, known as hypoxia, in tissues. In addition, nutrient availability, including the availability of glucose, fatty acids, and amino acids, can vary within the TME, impacting metabolic processes and energy production. The accumulation of metabolic waste products and alterations in pH can further contribute to a hostile TME, which can impair immune function and promote tumor progression.^23^ These factors, along with dynamic interactions within the TME, play crucial roles in influencing tumor proliferation and the effectiveness of antitumor immune responses.^24^

**Fig. 1:**
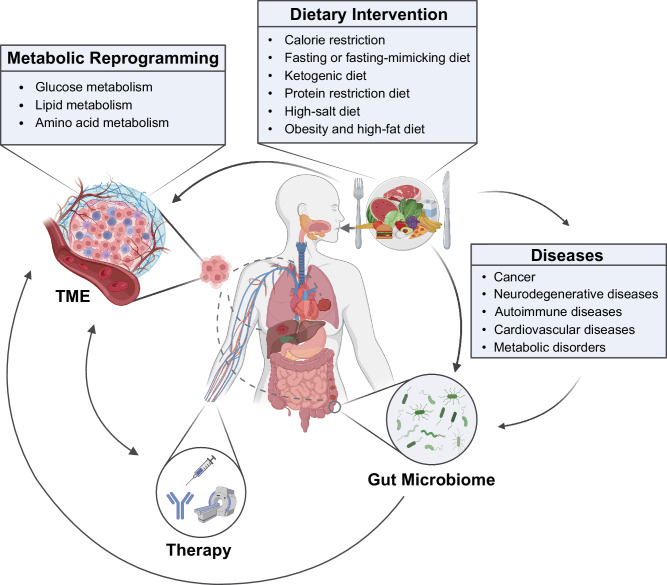
Overview of the relationship between dietary interventions and diseases. The cellular microenvironment, including the tumor microenvironment (TME), plays a crucial role in disease biology, and diet serves as a vital source of nutrients that can influence these microenvironments. Metabolic reprogramming, a prominent feature associated with disease progression, can affect cell metabolism and immune function. Dietary interventions, such as caloric restriction (CR), fasting-mimicking diet (FMD), and ketogenic diet (KD), can modulate the progression and treatment sensitivity of various diseases, including cancer. Additionally, dietary interventions can alter the composition and functional capacity of the gut microbiome, thereby indirectly influencing the progression and treatment of diseases. These direct and indirect effects of dietary interventions can influence metabolic reprogramming, modulate immune responses, and potentially enhance the clinical efficacy of treatments for various diseases. This figure was created with BioRender.com

As our understanding of the complex relationships between diet, metabolic reprogramming, and various diseases continues to evolve, it becomes increasingly evident that dietary components and patterns significantly influence disease risk, prevention, and progression. This review delves into the unique metabolic characteristics and nutrient availability of tumors. Furthermore, we investigate recent evidence and emerging trends concerning the effects of dietary interventions on both cancer and other diseases, underscoring the potential therapeutic benefits these dietary strategies may offer to a wide range of patients (Fig. 1).

## Metabolic characteristics and nutrient availability in the tumor

**Fig. 2:**
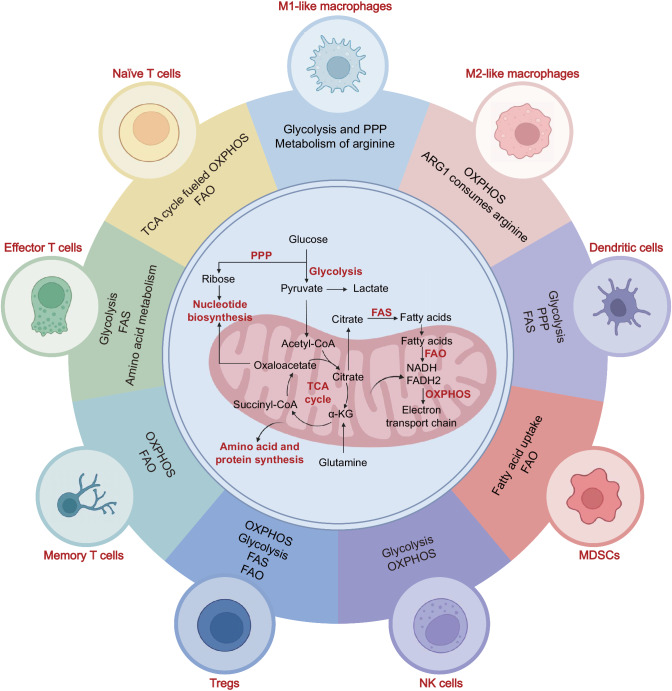
Major metabolic pathways associated with different immune cell subtypes within the tumor microenvironment (TME). Summary of the main metabolic pathways of immune cells, highlighting the distinctive metabolic characteristics and requirements of different subsets of immune cells. This figure was created with BioRender.com

Cellular metabolism encompasses a complex array of biochemical reactions that utilize specific nutrients, including carbohydrates, fatty acids, and amino acids. These nutrients are the primary sources for maintaining energy homeostasis and synthesizing macromolecules.^25^ Our focus here is on cancer metabolism, which differs from that in corresponding healthy tissues in terms of nutrient levels and metabolic demands.^26^ Within the TME, cancer cells can establish an immunosuppressive metabolic microenvironment by depriving immune cells of vital metabolites such as glucose and oxygen while also elevating the levels of mediators such as lactate and adenosine that limit the function of immune cells.^27^ Therefore, different subsets of immune cells undergo metabolic reprogramming in tumors, and specific nutrients are required for these metabolic programs.^28,29^ Generally, the metabolic programs that play vital roles in immune cells include glycolysis, the tricarboxylic acid (TCA) cycle, oxidative phosphorylation (OXPHOS), the pentose phosphate pathway (PPP), fatty acid oxidation (FAO), fatty acid synthesis (FAS) and the amino acid metabolic pathway^30^ (Fig. 2).

### Glucose metabolism

Glucose serves as a vital energy source, facilitating the functioning of immune cells. Once transported across the plasma membrane, glucose is metabolically processed via three distinct pathways: glycolysis, the PPP, and the TCA cycle. Glycolysis, which occurs in the cytosol, transforms glucose into pyruvate and lactate, simultaneously generating adenosine triphosphate (ATP). Under aerobic conditions, pyruvate is channeled into the TCA cycle, where OXPHOS occurs, yielding additional ATP. Moreover, glucose-6-phosphate, a derivative of glycolysis, fuels the PPP, culminating in the production of ribose-5-phosphate and nicotinamide adenine dinucleotide phosphate (NADPH). Recent research has indicated a marked disparity in energy consumption between immune cells in resting and activated states.^18^ Although glycolysis does not generate as many ATP molecules as OXPHOS, glycolysis produces ATP more rapidly, which is important to metabolically active immune cells.

Cancer cells are characterized by their rapid proliferation, primarily fueled by the consumption of glucose as an energy source. Intriguingly, these cells continue to rely on glycolysis for energy production even in the presence of ample oxygen, a phenomenon referred to as the “Warburg effect”.^31^ This unique phenomenon leads to glucose depletion and lactic acid (LA) accumulation in the microenvironment, ultimately inhibiting antitumor responses.^32^ High glycolytic rates in triple-negative breast cancer cells promote the infiltration of myeloid-derived suppressor cells (MDSCs) and suppress T-cell function, while suppressing glycolysis inhibits tumor colony-stimulating factor (CSF) expression and MDSC development.^33^ Cancer cells produce LA through glycolysis, which reduces the antitumor activity of CD8^+^ T cells and natural killer (NK) cells. However, the activation of LA metabolism pathways in regulatory T cells (Tregs) is increased, and these cells adapt to high-LA conditions.^34,35^ Furthermore, cancer cells can take advantage of immune cells by utilizing their metabolic byproducts. LA can shift tumor-associated macrophages (TAMs) from a proinflammatory (M1-like) to an anti-inflammatory (M2-like) phenotype in the TME. Notably, lactate-activated TAMs enhance cancer cell adhesion, migration, invasion in vitro, and promote metastasis in vivo.^36^

T cells play crucial roles in the TME. Upon activation, these cells undergo metabolic reprogramming, which subsequently yields diverse functional outcomes. Naïve T cells, which are metabolically quiescent, exhibit basic nutrient intake rates and low glycolysis rates. They primarily generate ATP through TCA cycle-fueled OXPHOS.^37^ The activation of specific membrane receptors triggers the differentiation of naïve T cells into effector T cells, also known as T_eff_ cells. This process is accompanied by a pronounced increase in both energy demand and biosynthetic activity within T_eff_ cells. In T_eff_ cells, the metabolic state is changed to increasingly rely on glycolysis, as these cells upregulate GLUT1, increase glucose intake.^38–41^ Simultaneously, this metabolic alteration benefits T_eff_ cells by reducing their reliance on oxygen for energy production, which enables them to maintain cytokine production and cytolytic activity even when they migrate into microenvironments within solid tumors that have low oxygen levels.^42^ In contrast to naïve and T_eff_ cells, memory T cells undergo a metabolic rewiring process that leads them to enter a quiescent state characterized by elevated OXPHOS rates compared to the glycolysis rate.^43^ Tregs, known for their suppressive function, exhibit decreased glycolysis rates and primarily rely on OXPHOS to support their function, while glycolysis is crucial for their migration.^44^ It has been reported that the Treg-specific transcription factor FOXP3 reprograms Treg metabolism by suppressing Myc expression and glycolysis while promoting OXPHOS and NAD(H) oxidation. This adaptation enables Tregs to be more adaptable to low-glucose and/or lactate-rich microenvironments.^45^

There are several other types of cells within the TME that exhibit distinct metabolic functions. In the case of NK cells, glycolysis and OXPHOS play important roles in maintaining their cytotoxicity, as indicated by the inhibition of these processes leading to diminished expression of IFNγ and Fas ligands.^46^ Researchers have shown that transcription factor-controlled glucose metabolism, specifically by sterol regulatory element-binding proteins (SREBPs), which conventionally control lipid synthesis, is essential for metabolic reprogramming in activated NK cells.^47^ Dendritic cells (DCs), on the other hand, rely on glycolysis and the PPP for energy production to sustain their function, including cytokine production, antigen processing and presentation, and the stimulation of T cells.^48^ Furthermore, different subsets of macrophages present distinct metabolic functions. M1-like macrophages predominantly utilize anabolic metabolism, specifically glycolysis and the PPP, to generate energy and synthesize cellular components, whereas M2-like macrophages are more reliant on OXPHOS, particularly through the enhancement of FAO.^49^

### Lipid metabolism

Lipids, such as fatty acids, triglycerides, cholesterol, phospholipids, and sphingolipids, play crucial roles as precursors to many important biological molecules.^50^ Lipids, including substances such as cholesterol and fatty acids that are widely distributed in organelles, are key components of internal cellular membranes. Moreover, lipids are essential biological molecules that provide energy during nutrient deficiency, participate in the synthesis of complex fat-containing substances, and aid in cellular signal transmission as second messengers.^51^ Lipids within the microenvironment profoundly influence the proliferation of cancer cells and regulate the functional activity of immune cells.

Cancer cells undergo metabolic reprogramming of lipids in the tumor niche. The activation of adipocytes triggers the lipolysis of stored triglycerides and secretion of fatty acids. Cancer cells can then take up these fatty acids to fulfill their lipid requirements for rapid growth.^52^ Research has also demonstrated that ovarian cancer cells stimulate membrane cholesterol efflux from TAMs, fostering an environment that promotes tumor growth by enhancing interleukin (IL)-4-mediated reprogramming and suppressing IFNγ-induced gene expression. The deletion of ABC transporters, responsible for cholesterol efflux, reversed the tumor-promoting functions of TAMs, leading to reduced tumor progression.^53^

Furthermore, elevated cholesterol levels in the microenvironment stimulate the expression of immune checkpoints, including PD-1, 2B4, TIM-3, and LAG-3, in T cells, driving T-cell exhaustion via the activation of the endoplasmic reticulum stress response.^54^ In contrast to the negative effects of reprogramming T-cell lipid metabolism on antitumor immunity, the inhibition of ACAT1, a pivotal enzyme responsible for cholesterol esterification in CD8^+^ T cells, results in elevated cholesterol levels in the plasma membrane. This increase subsequently amplifies TCR signaling and promotes antitumor activity. These findings highlight the complex mechanisms through which cholesterol regulates T-cell function.^55^

For efficient tumor antigen processing and presentation to T cells, activated DCs need high rates of cell surface or secretory protein biosynthesis, which is partly regulated by FAS-induced increases in cytokine production.^56^ T_eff_ cells depend mainly on FAS to support inflammatory cytokine secretion and proliferation, while naïve T cells and memory T cells maintain their basic functions by increasing the FAO rate.^57–59^ Although T_eff_ cells rely mainly on glycolysis for energy, CD8^+^ T cells that undergo enhanced FAO exhibit stable antitumor functions even under conditions of low glucose and oxygen levels. By promoting fatty acid catabolism, CD8^+^ T cells exhibit increased functionality, and the efficacy of immunotherapy in patients with melanoma can thus increase.^60^

While these studies indicate a positive influence of lipids on the functionality and metabolism of CD8^+^ T cells in the TME, it is important to note that alterations to T-cell lipid metabolism might attenuate their antitumoral effects. In obesity-related breast cancer murine models, the activation of STAT3 triggered an increase in FAO in CD8^+^ T cells, which suppressed glycolysis and weakened their tumor-suppressing ability.^61^ Moreover, enhanced lipid uptake and peroxidation can result in high oxidative stress, which leads to CD8^+^ T cell dysfunction. CD36, a fatty acid scavenger receptor, facilitates the incorporation of arachidonic acid into CD8^+^ T cells. This process subsequently triggers lipid peroxidation and ferroptosis, events that cumulatively attenuate the antitumor immune response and reduce the efficacy of immunotherapy.^62–64^

Lipid metabolism also plays an active role in regulating Treg function. Fatty acid synthase (FASN)-mediated FAS contributes to the proliferation and maturation of Tregs, and FAO provides the energy crucial for Treg infiltration into the TME.^65^ Research has shown that OX40 plays a role in modifying the lipid composition of Tregs, leading to the proliferation of OX40^+^ Tregs in the TME. This effect is achieved through increased FAS expression and glycolysis rate in Tregs.^66^ CD36, via the peroxisome proliferator-activated receptor-β (PPAR) signaling pathway, maintains the mitochondrial fitness of Tregs, promoting Treg viability and inhibitory functions.^67^ SREBPs have been found to show increased activity in Tregs that infiltrate tumors. Inhibiting FAS and metabolic signaling by targeting SREBPs has been shown to effectively activate the antitumor immune response without causing autoimmune toxicity. When the SREBP-SCAP axis was inhibited, in addition to tumor growth attenuation, immunotherapy effectiveness was boosted. These findings suggest that SREBPs may be promising targets for cancer therapy.^68^

High expression of FASN in TAMs promotes the accumulation of fatty acids, leading to enhanced tumor immune tolerance via the FAO pathway.^69^ Notably, lipid metabolism differs between M1-like and M2-like macrophages. M1-like macrophages prevalently engage the FAS pathway, while M2-like macrophages predominantly utilize the mitochondrial FAO pathway for their bioenergetic demands.^70,71^ Receptor-interacting protein kinase 3 (RIPK3), which is crucial for necroptosis, is found to be diminished in hepatocellular carcinoma (HCC)-associated macrophages, leading to inhibited caspase1-mediated cleavage of PPAR, a process vital for enhancing fatty acid metabolism, including FAO. This metabolic shift results in increased accumulation and polarization of M2-like macrophages in the TME, contributing to accelerated HCC growth.^72^

MDSCs also exert a substantial influence in suppressing antitumor immunity in the microenvironment, and they can be categorized into monocytic MDSCs (M-MDSCs) and granulocytic MDSCs (PMN-MDSCs).^73^ Tumor-infiltrating MDSCs increase fatty acid uptake and induce FAO.^74^ The accumulation of lipids in MDSCs increases oxidative metabolism, resulting in MDSC acquisition of an immunosuppressive and anti-inflammatory phenotype.^75^

### Amino acid metabolism

Amino acids are the primary substrates for protein biosynthesis, and recent evidence emphasizes the critical role of amino acid availability and metabolism in the regulation of antitumor immunity.

Glutamine is the most abundant amino acid and a crucial energy substrate, as well as an important nitrogen and carbon donor for various biosynthetic precursors.^76^ T_eff_ cells require higher levels of glutamine than naïve T cells due to their rapid proliferation and demand for sufficient raw materials for macromolecule synthesis and cytokine secretion.^77^ Cancer cells have been shown to exhibit the highest glutamine uptake capacity and consume most of the glutamine in the microenvironment.^76^ In turn, elevated glutamine consumption by cancer cells diminishes the glutamine supply necessary for T cells, consequently impeding the antitumor immune response.^78^ In the microenvironment, cancer cells consume glutamine to synthesize γ-aminobutyric acid (GABA) via glutamate decarboxylase 1 (GAD1). By activating the GABA_B_ receptor, GABA inhibits GSK-3β activity, which enhances β-catenin signaling, promoting cancer cell proliferation while suppressing intratumoral infiltration of CD8^+^ T cells.^79^ Furthermore, elimination of glutaminase, a vital enzyme for glutamine metabolism, within tumor cells stimulates T-cell activation and augments the efficacy of antitumor immune responses. The compound V-9302, an inhibitor of the glutamine transporter, selectively impedes glutamine uptake in cancer cells while simultaneously enhancing both glutamine assimilation and glutathione synthesis in T_eff_ cells, ultimately enhancing their function.^80^

Tryptophan is another essential amino acid. Following its entry into eukaryotic cells via the transport proteins SLC1A5 or SLC7A5, tryptophan is primarily subjected to three primary metabolic pathways: incorporation into protein synthesis, metabolism via the kynurenine (Kyn) pathway, or conversion through the serotonin pathway.^81^ Notably, a substantial fraction of tryptophan is directed through the Kyn pathway, culminating in the production of a suite of metabolites with significant physiological implications.^82^ Tryptophan plays a crucial role in determining the strength and effectiveness of the T cell response by affecting its availability in the microenvironment.^83^ However, within the tumor niche, cancer cells, MDSCs, TAMs, suppressive DCs, and cancer-associated fibroblasts, among other cell types, exhibit upregulated expression of indoleamine 2,3-dioxygenase (IDO), which metabolizes tryptophan into suppressive kynurenine to promote Tregs and suppress CD8^+^ T cell function.^84–86^ Most cancer cells overexpress IDO, and the level of kynurenine in the microenvironment is associated with poor prognosis in multiple solid and hematological malignancies.^87^ Kynurenine has been found to bind to the aryl hydrocarbon receptor (AHR) in naïve CD4^+^ T cells, which promotes Treg differentiation.^87^

An additional metabolite generated through the Kyn pathway is the essential redox cofactor nicotinamide adenine dinucleotide (NAD+), a molecule of fundamental importance for the maintenance of cellular homeostasis.^88^ In particular, cancer cells heavily depend on NAD+ to promote metabolic reprogramming and meet higher demands for ATP. Elevated NAD+ levels have been demonstrated to promote the proliferation of cancer cells.^89^ Although the majority of studies suggest that an increase in NAD+ drives cellular proliferation, prior investigations have proposed that a decrease in NAD+ levels can lead to genomic instability, subsequently instigating liver tumorigenesis.^90^ Moreover, tryptophan metabolism mediated by IDO affects not only the Kyn pathway but also other pathways, such as the purine, nicotinamide, and pyrimidine metabolism pathways, ultimately leading to decreased T-cell function.^91^ In addition to IDO, another enzyme, tryptophan 2,3-dioxygenase (TDO), is involved in tryptophan catabolism. High TDO expression has been shown to impair T-cell antitumor immunity and to be correlated with poor clinical prognosis. Suppressing TDO expression can increase the antitumor efficacy of immune checkpoint inhibitors (ICIs).^92^

In addition to the aforementioned amino acids, other amino acids play crucial roles in regulating tumor metabolism. T-cell proliferation relies heavily on arginine consumption. L-arginine supplementation has been shown to facilitate the metabolic shift from glycolysis to OXPHOS, enhancing T-cell survival and boosting antitumor responses of CD8^+^ tumor infiltrating lymphocytes (TILs).^93^ Notably, the functional differences resulting from TAM polarization partially depend on arginine metabolism. In macrophages with the M1-like phenotype, arginine is converted into nitric oxide (NO) and citrulline via inducible nitric oxide synthase (iNOS), and this anabolic pathway is closely associated with macrophage cytotoxicity and antitumor effects. Conversely, in macrophages with the M2-like phenotype, arginine is hydrolyzed to yield ornithine and urea through arginase 1 (Arg1).^94^ This metabolic shift affects arginine availability, which in turn impacts the activation and proliferation of T cells and NK cells, leading to immune suppression within the microenvironment. Notably, Arg1 expression in MDSCs contributes to arginine depletion in the microenvironment, further inhibiting T-cell antitumor function and reducing their survival.^95,96^ In addition, depletion of cystine and cysteine is also linked to the immunosuppressive effect of MDSCs. T cells are unable to synthesize the essential amino acid cysteine from substances such as cystine or methionine, necessitating its import from external sources for their functionality.^97^ MDSCs import cystine but do not release cysteine, thus the levels of cysteine in the microenvironment are regulated, inhibiting T-cell activation.^98^ Asparagine is another amino acid that significantly boosts CD8^+^ T-cell activation and antitumor responses. Restricting dietary asparagine or inhibiting its uptake impaired T-cell activation and differentiation into memory-like cells.^99^ Cancer cells consume higher levels of methionine due to increased expression of its transporter (SLC43A2), which inhibits methionine metabolism and function in CD8^+^ T cells by altering histone methylation patterns.^100^

### Organ-specific metabolic profiles

Understanding the metabolic differences between various organs is critical for developing targeted therapeutic strategies in cancer treatment. Each organ has unique metabolic demands and pathways that can be dysregulated in cancer, leading to distinct metabolic profiles for different types of tumors.^101^ This organ-specific metabolic reprogramming plays a key role in cancer progression and survival, and its understanding could be leveraged for therapeutic benefits.

Consider primary brain tumors as an example. These tumors, often found nestled within the intricate neural networks of the brain, exhibit a remarkable metabolic flexibility.^102^ They are known to express elevated levels or alternative isoforms of glycolytic enzymes, a trait that points towards a potential therapeutic opportunity.^103^ Specifically, the therapeutic strategy of glucose deprivation could selectively starve brain tumor cells while sparing healthy neurons, which are capable of surviving on alternative fuels such as ketone bodies.^104^ Similarly, HCC cells undergo a significant metabolic shift from glucose production (a state known as gluconeogenesis) to glucose usage.^105^ HCC cells also exhibit a marked increase in amino acid metabolism, particularly in the metabolism of glutamine.^106^ Additionally, studies have shown that HCC cells often exhibit abnormal lipid accumulation, increased FAS, and enhanced cholesterol metabolism. These changes contribute to the aggressive and metastatic behaviors of HCC.^107^

Moreover, hormone-sensitive tissues such as the breast, endometrium, and prostate also exhibit significant metabolic fluctuations in response to hormone levels.^101^ Hyperactivation of the PI3K pathway, a lipid kinase that promotes proliferation and nutrient uptake in response to growth signals, has been implicated in breast and endometrial cancers, providing a possible mechanism for hormonal therapy evasion.^107^ This pathway could be a potential target for therapeutic interventions, particularly in hormone therapy-resistant cancers.

In summary, understanding organ-specific metabolic profiles and their dysregulation in cancer can open up new avenues for targeted cancer therapy. By exploiting these unique metabolic dependencies of tumors, more effective and personalized treatment strategies can be developed.

## Targeted dietary interventions and mechanistic insights into their impact on cancer

Understanding the metabolic pathways of glucose, lipids, and amino acids lays a crucial foundation for exploring the effects of various dietary restrictions. Macronutrients, including carbohydrates, fats, and proteins, are the primary sources of energy for our bodies, and they each follow distinct metabolic pathways. By manipulating the relative intake of these macronutrients, we can influence the metabolic pathways they utilize and thereby exert control over our systemic metabolism. This concept forms the basis for various dietary restrictions and special diets, such as caloric restriction (CR), fasting or fasting-mimicking diet (FMD), ketogenic diet (KD), high-fat diet (HFD), or amino acid-defined diet. Moreover, high-salt diet (HSD), although not directly involving macronutrients, is noteworthy due to its potential impact on tumor biology. Therefore, an in-depth discussion on the role of HSD in cancer research and treatment is included in our exploration.

The connections between various dietary patterns and cancer risk are likely rooted in several biological mechanisms, such as inflammation and immune function; specific factors, such as the gut microbiota and their metabolites; unfavorable events, such as certain epigenetic changes and metabolic or hormonal disruptions; and stress, such as oxidative stress.^108^ Alterations in dietary composition impact not only the availability of nutrients within tumor cells but also the surrounding microenvironment, thereby offering potential opportunities to impede tumor growth^109^ (Table 1).

**Table 1:** Preclinical studies supporting dietary interventions in cancer

Dietary intervention	Cancer type	Results	References
Calorie restriction	Breast cancer	Ly6C^+^-expressing memory T cells (CD4^+^Ly6C^+^)↑, Ly6C^+^CD8^+^ cells↑, CD103^+^CD4^+^ cells↑, CD103^+^CD8^+^ cells↑ FOXP3^+^CD8^+^ Tregs↓, MDSCs↓, PMN-MDSC↓, TAMs↓	^118^
		(1) ER (energy restriction) vs. AL: the expression of *Aicda*, *Pdcd1*, *Ifng*, *Foxp3*, and *Ido1*↓ (2) PA (physical activity) + ER vs. SED (sedentary)+ AL: (i) For tumors at equal size: CD8^+^ T cells↑, CD4^+^ T cells↑, CD8/total MDSC ratio↑, MSDCs↓, M-MSDCs↓ (ii) For tumors at day 35 post-tumor implantation: CD8^+^ T cells↑, CD8/total MDSC ratio↑, MSDCs↓, M-MSDCs↓, PMN-MSDCs↓	^124^
	Colorectal cancer	CD8^+^ T cells↑, IFNγ^+^CD8^+^ T cells↑	^290^
Fasting-mimicking diet	Breast cancer	CTLA-4^+^ Tregs↑, PD-1^+^ Tregs↑ Myeloid cells↓, M2-like macrophages↓, PMN-MDSCs↓, M-MDSCs↓, PD-L1^+^ PMN-MDSCs↓, PD-L1^+^ M-MDSCs↓	^236^
Alternate day fasting	Colorectal cancer	TAMs↓, M2-like macrophages↓	^137^
Ketogenic diet	Colorectal cancer	Macrophages↑, M2 to M1 TAM polarization↑ Lactate↓	^151^
		Hopx activation↑, colonic crypt cell proliferation↓, tumor growth↓	^147^
	Non-small cell lung cancer	Per↑, AMPK activation↑, SIRT1↑, tumor cell apoptosis↑, tumor cell growth↓	^148^
	Neuroendocrine cancer	PI3K-Akt-mTOR signaling↓, tumor growth↓	^149^
	Glioma	CD4^+^ T cells↑, CD4^+^ T cells/Tregs ratio↑, IFNγ^+^CD8^+^ T cells↑, TNF^+^CD8^+^ T cells↑, IL-2^+^CD8^+^ T cells↑, cytotoxic capability of CD8^+^ T cells↑, IFNγ^+^ NK cells↑, TNF^+^ NK cells↑ PD-1^+^CD8^+^ T cells↓, CTLA-4^+^CD8^+^ T cells↓, IL-10^+^ Tregs↓	^152^
	Colorectal cancer Adrenocortical cancer	Tumor cell ferroptosis↑, cachexia onset↑, overall survival↓	^156^
Dietary restriction of protein/80% methionine-restricted diet	Prostate cancer	M1-like macrophages↑, M1-like macrophages linked proteins (CXCL11/I-TAC, IL-1α, IL-1β, IL-12p40, M-CSF, and IL-17A) ↑, CD8^+^ T cells↑, granzyme B^+^CD8^+^ T cells↑ M2-like macrophages↓, PMN-MDSCs↓, M-MDSCs↓, M2-like macrophages linked proteins (C-reactive protein, FGF acidic, IL-33, leptin, and MMP9) ↓	^163^
Dietary methionine restriction	Colorectal cancer	CD8^+^ T cells↑, GZMB^+^CD8^+^ T cells↑, IFNγ^+^CD8^+^ T cells↑ L-cystathionine (LCYH)↓, SAM↓, 5’-methylthioadenosine (MTA)↓, S-adenosylhomocysteine (SAH)↓, glutathione (GSH)↓, L-methionine (Met)↓, homoserine↓	^162^
Dietary serine and glycine restriction	Intestinal cancer Lymphoma	Anti-oxidant response↑	^161^
Low-protein diet	Breast cancer	mTORC1 signaling↓, TFEB↑, TFE3↑, mTORC1↑, tumor-associated macrophages↑	^164^
	Breast cancer Melanoma	IGF-1↓	^157^
Low-protein isocaloric diet	Melanoma	NK cells↑, CD3^+^ cells↑, CD8^+^ cells↑	^165^
High-protein diet	Bladder cancer	Urinary urea↑, intracellular deposition of ammonia↑, tumor growth↓	^166^
		Overactivation of CRP, MCPT2, MCPT9, EPXH2, SERPING1, SRGN, CDKN1C, CDK6, CCNB1, PCNA, BAX, MAGEB16, SERPINE1, HSPA2, and FOS	^167^
High-salt diet	Melanoma Lung cancer	The expression of *Tnfα*, *Ifnγ*, and *Nos2*↑ Suppressive function of MDSCs↓	^184^
	Breast cancer Melanoma	IL-12p40↑, ICAM-1↑, IFNγ↑, TNFα↑, macrophages↑, M-MDSCs differentiation into antitumor macrophages↑, functions of PMN-MDSCs switch from immunosuppressive to proinflammatory and antitumor↑, CD4^+^ T cells↑, CD8^+^ T cells↑, IFNγ^+^CD4^+^ T cells↑, IFNγ^+^CD8^+^ T cells↑, Th17 cells↑, TNFα^+^ Th17 cells↑ IL-6↓, IL-10↓, GM-CSF↓, MDSCs↓, M-MDSCs↓, Tregs↓	^185^
	Melanoma	NK cells↑, CD107a^+^ NK cells↑, IFNγ^+^ NK cells↑, *Bifidobacterium*↑ PD-1^+^ NK cells↓, CTLA-4↓, PD-1↓	^183^
	Breast cancer	Hyperosmotic stress↑, lung metastasis↑	^187^
		Th17 cells↑, the expression of *Il17f, Il21, Il22 and Rorγt*↑	^190^
		γENaC mediated chronic inflammatory response↑, RNS/ROS↑, IL-6↑, TNFα↑, tumor growth↑	^191^
High-fat diet	Nonalcoholic steatohepatitis and hepatocarcinogenesis	Hepatic unconventional prefoldin RPB5 interactor (URI) ↑, Th17↑, IL-17A↑ Neutrophil infiltration into white adipose tissue, causing insulin resistance and release of fatty acids	^199^
	Helicobacter-induced chronic gastric inflammation and gastric carcinogenesis	Immature myeloid cells↑, CD4^+^ T Cells↑, IL-17A↑, granulocyte macrophage colony-stimulating factor↑, phosphorylated STAT3↑	^200^
	Colorectal cancer	Myeloid cells↑, MDSCs↑, TAMs↑, IL-2^+^CD8^+^ T cells↑, triglyceride↑, diglyceride↑ CD8^+^ T cells↓, leukocyte/tumor cell ratio↓, CD8^+^ T cells/tumor cell ratio↓, Ki67^+^CD8^+^ T cells↓, CD8^+^/Treg ratio↓, ICOS^+^CD8^+^ T cells↓, PD-1^+^CD8^+^ T cells↓, GZMB^+^CD8^+^ T cells↓, fatty acid↓	^207^
		PD-1^high^CD4^+^ T cells↑, PD-1^high^CD8^+^ T cells↑ CD4^+^ T cells↓, CD8^+^ T cells↓, central memory CD4^+^ T cell↓, effector memory CD4^+^ T cell↓, CD107a^+^CD4^+^ T cells↓, CD107a^+^CD8^+^ T cells↓, TNFα^+^CD4^+^ T cells↓, IFNγ^+^CD4^+^ T cells↓, TNFα^+^CD8^+^ T cells↓, IFNγ^+^CD8^+^ T cells↓	^209^
		IL-6↑, M2-like TAMs↑, CCL20↑, B cells↑, Tregs↑, αβT cells↑, γδT cells↑	^203^
		Total macrophages↑, M2-like macrophages↑ M1-like macrophages↓	^296^
		EVs↑, YAP signaling↑, CYR61↑, M2-like macrophages↑, liver metastasis↑	^212^
	Oral squamous cell carcinoma	CD45^+^ cells↑, myeloid cells↑, MDSCs (mainly PMN-MDSCs) ↑, CCR1^+^ PMN-MDSCs↑, Arg1^+^ MDSCs↑, the expression of *Arg1* and *S100a9*↑ T cells↓	^205^
	Prostate cancer	MDSCs↑, M2/M1 macrophage ratio↑, the expression of *Il6, Il1b, Il13*, and *Il17a*↑, pSTAT3^+^ cells/tumor cells ratio↑	^202^
		SREBP prometastatic lipogenic program↑, lipid↑	^213^
	Breast cancer	MDSCs↑, effector CD8^+^ T cells↑, PD-1^+^CD8^+^ T cells↑, Ki-67^+^CD8^+^ T cells↑, IFNγ^+^CD8^+^ T cells↑, apoptotic CD8^+^ T cells↑, Fas^+^CD8^+^ T cells↑, PMN-MDSCs↑, the expression of MDSC-related cyto/chemokines (*Il1b*, *Cxcl1*, *Cxcl3*, *S100a8*, and *Csf3*) ↑, CXCL1↑, per cell expression of FasL in PMN-MDSCs↑ naïve CD8^+^ T cells↓, IFNγ↓, Bcl-2^+^CD8^+^ T cells↓	^206^
		Overexpression of nitric oxide synthase, NO↑, recruitment of macrophages↑	^201^
		Palmitate↑, acetyl-CoA↑, lysine acetyltransferase 2a↑, nuclear factor-kappaB subunit p65 acetylation↑, lung and liver metastasis↑	^211^
		CD36 palmitoylation↑, MUFAs intake↑, palmitate-induced lipotoxicity↓	^215^
	Colorectal cancer	Valine↑, leucine↑ CD45^+^ cells↓, CD8^+^ T cells↓, IFNγ^+^CD8^+^ T cells↓, IFNγ^+^TNF^+^CD8^+^ T cells↓, GZMB^+^CD8^+^ T cells↓, Ki67^+^CD8^+^ T cells↓, PD-1^+^CD8^+^ T cells↓, CD98^+^CD8^+^ T cells↓, pS6^+^CD8^+^ T cells↓, glutamine↓, arginine↓, ornithine↓, kynurenic acid↓	^208^
	Melanoma	CD45^+^ cells↓, CD8^+^ T cells↓, CD4^+^ T cells↓, NK cells↓, CD49d^+^CD8^+^ T cells↓, CXCR3^+^CD8^+^ T cells↓, the expression pf *Cxcl9* and *Cxcl10*↓, IFNγ^+^TNF^+^CD8^+^ T cells↓, lipidtox^+^CD8^+^ T cells↓	
	Pancreatic cancer	TAMs↑, IL-1β↑, IL-4↑, IL-5↑, IL-2↑	^204^
		CD45^+^ cells↑, myeloid cells↑, MDSCs↑, tumor-associated neutrophils↑, IL-1β↑	^210^
	Melanoma	PD-1^+^CD8^+^ T cells↑, Tim3^+^CD8^+^ T cells↑, Lag3^+^CD8^+^ T cells↑, expression of *Cpt1a*↑ Ki67^+^CD8^+^ T cells↓	^246^
High-cholesterol diet	Colorectal cancer	IL-1β↑, IL-6↑, TNFα↑, macrophages↑, NLRP3 inflammasome activation↑	^217^
		Macrophages↑ IFNγ^+^CD8^+^ T cells↓	^218^
	Hepatocellular carcinoma	NK cells↑, NK cells↑, effector function of NK cells↑, CD8^+^ T cells↑	^221^
Fish oil high-fat diet (vs. cocoa butter high-fat diet)	Breast cancer	ROS production in TAMs↑ TAMs↓	^222^
Fish oil high-fat diet (vs. corn oil high-fat diet)	Prostate cancer	M1-like macrophages↑ M2-like macrophages↓, the expression of *CD206*, *Arg1*, *TNFα*, *CCL2*, *CCL22*, *MMP-9*, *VEGF*↓	^223^
Safflower oil high-fat diet (vs. olive oil high-fat diet)	Breast cancer	CD8^+^ T cells↓, CD4^+^ T cells↓, TNFα^+^CD8^+^ T cells↓, TNFα^+^CD4^+^ T cells↓	^224^
High-fiber diet	Lymphoma	DCs↑, cDC1↑, Ifnb1^+^ monocytes↑, Xcl1 on NK cells↑	^301^
	Colorectal cancer	DCs↑, monocytes↑ Macrophages↓	

### Calorie restriction

Effective CR is a dietary intervention that reduces energy intake by approximately 15–30% while maintaining a balanced proportion of macronutrients and preventing malnutrition.^110^ CR has been shown to prolong life and reduce age-related diseases, including cancer, in experimental models.^111^

Although the antitumor effect of CR has been confirmed, the underlying mechanism remains unclear. Nonetheless, it is believed that the tumor-inhibiting effect is partially mediated by several biological changes, such as increased apoptosis rates in cancer cells, decreased circulating blood glucose levels, inhibited insulin-like growth factor 1 (IGF-1) signaling, reduced insulin levels, and mediators that regulate metabolic pathway activation and inhibit angiogenesis.^112^ In particular, controlling IGF-1 signal transduction is a critical component underlying the antitumor effects of CR. The IGF-1 signaling pathway is frequently activated in cancer cells, and it shifts metabolic resources toward growth and proliferation. Therefore, the reduction in IGF-1 levels in response to CR leads to attenuated tumor growth and progression.^113^ The impact of CR on cancer is also interconnected with mutations and oncogenic pathways. A study showed that CR results in a reduction of insulin levels, thereby diminishing tumor PI3K signaling.^114^ CR has also been found to suppress xenograft tumor growth by upregulating the aldolase A (ALDOA)/DNA-PK/p53 pathway, with ALDOA acting as a potential oncogene that can also activate the tumor suppressor p53.^115^ Moreover, CR has been shown to modify the cancer stem cell (CSC) phenotype, reducing their carcinogenic and metastatic potential. Notably, in MMTV-ErbB2 transgenic mice, the CSC subpopulation was most affected by CR, as shown by a reduction of luminal cells (CD24^high^/CD49f^low^), putative mammary reconstituting unit subpopulations (CD24^high^/CD49f^high^) and luminal progenitor cells (CD61^high^/CD49f^high^). These effects were largely attributed to the concurrent inhibition of estrogen receptor and ErbB2 signaling.^116^

CR has been shown to shape the TME in several ways, including through the specific reduction in the number of TAMs, increase in the formation of CD8^+^ cytotoxic T cells and memory T cells, and negative modulation of immunosuppressive Treg cell activity and immunosuppressive cytokine levels.^117^ Additionally, CR promotes favorable changes in the immune signature, providing enhanced protection against tumor growth and metastasis, possibly in part by remodeling the TME. In mice, no impact of a CR diet was observed on the number of CD4^+^ or CD8^+^ cells in the TME; however, the cytotoxic killing potential of these cells was elevated. Notably, higher expression of CD103^+^, a marker of crucial tissue-resident memory T cells that possess enhanced cytotoxic capacity and can contribute to tissue protection against tumor cell invasion, was found. Additionally, a downward trend in the frequency of Tregs was observed, and a significant reduction in the total number of MDSCs was detected.^118^ Hence, it was concluded that CR not only inhibits cancer cell proliferation but also helps maintain antitumor immunity.

Furthermore, research has shown that fasting, CR, and caloric restriction mimetics (CRMs) can promote T-cell-mediated tumor cytotoxicity, alter NK cell function, and potentially trigger immunogenic cell death, thereby stimulating cancer immunosurveillance pathways.^119^ CRMs are pharmacological agents or natural compounds that imitate the biochemical effects of CR by reducing the lysine acetylation rates of cellular proteins.^120^ Examples of CRMs include hydroxycitrate (an inhibitor of ATP citrate lyase), spermidine (an inhibitor of EP300 acetyl transferase activity), and resveratrol (an activator of sirtuin-1 deacetylase activity).^121^ Treatment with CRMs has been found to decrease the concentration of free IGF-1, promote autophagy in cancer cells, and improve the antitumor immune response, resulting in a reduction in tumor growth when combined with immunogenic chemotherapeutics.^119^ CRM hydroxycitrate has been found to stimulate autophagy in U2OS osteosarcoma cells in vitro, thereby increasing antitumor immunosurveillance and reducing tumor mass in mice with autophagy-competent mutant KRAS-induced lung cancers.^122^ Moreover, in vitro treatment with resveratrol inhibits mitochondrial respiration in breast cancer cell lines through a SIRT1-dependent mechanism, diminishes the expression of markers associated with breast CSCs, and promotes their differentiation.^123^ Collectively, these findings suggest that CRMs may enhance antitumor immunosurveillance in preclinical models.

Moderate physical activity, energy restriction, and their combination can also affect tumor growth. In fact, the combined effects of moderate physical activity and 10% energy restriction (PA + ER) have been shown to significantly delay primary tumor growth, reduce spontaneous metastases, and prolong survival. These effects on tumor progression and survival are accompanied by beneficial changes in immune cell infiltrates within the microenvironment. Specifically, the PA + ER combination leads to an increase in the percentage of CD8^+^ T cells and a decrease in the percentage of total MDSCs and MDSC subsets within tumors.^124^

Nevertheless, it is crucial to emphasize that there are established nutritional recommendations for cancer care, and the weight loss or reduction in protein intake often associated with CR may conflict with these guidelines.^125^ These dietary practices could exacerbate the risk of malnutrition, sarcopenia, fatigue, delayed wound healing, and impaired immunity, particularly in cancer patients who are already at an increased age-associated risk for these conditions.^126^ Therefore, while exploring dietary interventions for cancer treatment, the potential adverse effects on overall patient health and nutritional status must be carefully considered.

### Fasting or fasting-mimicking diet

In addition to CR, alternative approaches such as intermittent fasting (IF), including short-term fasting (STF), intake of an FMD, and time-restricted feeding (TRF), which limits food consumption to a specific time window each day, are being condisered.^127,128^ The term “fasting” has a broad definition, encompassing a range of eating patterns, including complete and voluntary deprivation of food with no restriction on drinking water.^129^ An FMD is based on a regimen of low-calorie and low-protein foods that mimics the effects of fasting but induces fewer side effects. This approach retains the benefits of traditional fasting methods while minimizing their potential drawbacks.^130^

Fasting or intake of an FMD can cause various metabolic changes, including alterations in the systemic levels of hormones and growth factors such as insulin, glucagon, growth hormone, IGF-1, glucocorticoids or adrenaline.^131^ In response to these changes, normal cells activate protective mechanisms against stress and toxic insults, thereby reducing their metabolic requirements and cell division rate. On the other hand, because fasting or FMDs reduce tumor growth-promoting nutrients and factors, cancer cells struggle to manage metabolite deprivation and thus develop greater sensitivity to cancer therapies.^132^ In obesity-driven postmenopausal cancer mouse models, TRF was shown to delay the onset of tumors and reduce lung metastasis. Moreover, TRF was found to increase systemic insulin sensitivity and decrease hyperinsulinemia. Importantly, TRF could also restore the circadian rhythm of gene expression within tumors while attenuating both tumor growth and insulin signal transduction.^133^ Fasting can cause an “anti-Warburg effect” by reducing aerobic glycolysis and glutaminolysis while increasing OXPHOS uncoupled from ATP synthesis.^134^ In cancer cells, OXPHOS increases reactive oxygen species (ROS) production and leads to oxidative stress, activation of p53 signaling and DNA damage, particularly when combined with chemotherapy or other cancer therapies.^135^ Therefore, the unique metabolic vulnerabilities of cancer cells, which differ from those of normal cells, can be strategically targeted to develop novel and effective therapeutic interventions. According to a recent study, the combination of chemical treatment with an FMD reduces the expression of heme oxygenase-1 (HO-1), which is a stress-responsive enzyme that protects cancer cells against oxidative damage and apoptosis in vivo. Interestingly, this combination treatment resulted in upregulated HO-1 expression in normal cells. The downregulation of HO-1 production in cancer cells, in part, facilitated FMD-induced chemosensitization of cancer cells by boosting CD8^+^ TIL-dependent cytotoxicity, which was possibly facilitated by decreased Tregs.^136^ A separate study conducted with mouse models of colon cancer indicated that alternate day fasting for 2 weeks triggered autophagy in cancer cells, which in turn downregulated CD73 expression. As a result, the production of immunosuppressive adenosine in cancer cells was reduced, ultimately preventing macrophages from acquiring an M2 immunosuppressive phenotype.^137^

Clinical experiments have suggested that intake of an FMD can induce metabolic changes and increase antitumor immunity in cancer patients. In fact, the final outcomes of an FMD-treated clinical trial (NCT03340935) demonstrated that a severely calorie-restricted, five-day FMD regimen was well tolerated and resulted in substantial systemic metabolic changes in patients with different tumor types who were concurrently receiving antitumor therapies.^138,139^ In another clinical trial called DigesT (NCT03454282), a five-day FMD regimen was found to broadly reshape intratumor immunity in breast cancer patients. Specifically, the FMD was shown to promote the infiltration of activated and cytotoxic immune cell populations, including total and activated intratumoral CD8^+^ T cells, M1-like macrophages, aDCs, and NK cells. These changes were paralleled by an increase in immune signatures associated with improved clinical outcomes in cancer patients.^138^

### Ketogenic diet

A KD comprises a high-fat component, very low carbohydrate levels, and low to moderate protein levels, as explained in a recent study.^140^ A traditional KD is typically formulated at a 4:1 ratio of fat:carbohydrate plus protein.^141^ In this classical formulation, 80–85% of calories are derived from fat, 10–15% from protein, and less than 5% from carbohydrates.^142^ A KD is known to be effective at treating epilepsy, lowering glucose levels, and producing ketone bodies in vivo.^143^ There is increasing evidence to support the use of KD as a potential tumor treatment or prevention method, either as a standalone approach or in combination with other medicines.^144^

The Warburg effect indicates that lower intratumoral glucose levels can impede tumor growth, which can be achieved through pharmacological intervention and dietary changes such as a KD. Cancer cells, unable to utilize ketone bodies produced by KD for energy due to their aberrant mitochondrial function and diminished enzyme activity, can essentially be “starved” of glucose. Hence, KD emerges as a potentially promising strategy for cancer prevention.^145^ One of the primary ways in which a KD potentially promotes potential anticancer effects is by increasing the levels of β-hydroxybutyrate (β-HB), which is the most abundant ketone body.^146^ For instance, β-HB has been proven to inhibit CRC by activating the transcriptional regulator Hopx through the surface receptor Hcar2, thereby reducing the proliferation of colonic crypt cells and suppressing tumor growth.^147^ Another antitumoral effect of KD is upregulating the expression of the circadian clock gene Per (Period) by activating AMPK and upregulating SIRT1 (Sirtuin1), resulting in enhanced apoptosis and growth delay in tumor cells.^148^ KD also decreases insulin-regulated PI3K-Akt-mTOR signaling, which is overactivated in pancreatic neuroendocrine tumors (PanNETs), resulting in decreased blood glucose levels and a suppressive effect on the development and progression of PanNETs.^149^

Emerging evidence suggests that a KD may be a valuable clinical tool to enhance T-cell-mediated antitumor immune responses. In vitro and in vivo studies have shown that KD intake markedly increased the specific responses of human T cells, resulting in enhanced CD4^+^, CD8^+^, and Treg capacity, as well as augmented T memory cell formation. Under conditions of KD intake, CD8^+^ T cells undergo metabolic reprogramming to rely on OXPHOS in response to increased ketone bodies, leading to enhanced cellular energy and respiratory reserve, potentially improving their functionality.^150^ In addition, KD intake prevented the progression of colon tumors by inducing tumor cell oxidative stress, inhibiting MMP-9 expression, and promoting M2 to M1 TAM polarization.^151^ In a mouse model of malignant glioma, KD feeding led to significantly enhanced innate and adaptive tumor-specific immune responses. Mice fed a KD showed increased cytokine production (IFNγ, TNF, and IL-2) and greater tumor-reactive CD8^+^ T-cell cytotoxicity. Moreover, the mice maintained on a KD presented with a higher number of immune cells and a higher ratio of CD4^+^ T cells to Tregs, while the functionality of the Tregs was weakened. Feeding mice with the KD resulted in a noteworthy decrease in the expression of immune inhibitory receptors (PD-1 and CTLA-4) on CD8^+^ TILs, as well as a reduction in the expression of inhibitory ligands (CD86 and PD-L1) on cancer cells.^152^ These findings suggest that a KD has the potential to attenuate tumor-induced T-cell suppression by decreasing the population of cells susceptible to the inhibitory PD-1 pathway.

Although KD has shown various potential benefits to tumor patients with its promising effects of inhibiting tumor cell growth and activating immune response, there is still limitation in its clinical application owing to its inevitable side effects.^153^ It should be considered that KD also presents some risks, as they are typically high in saturated fats and may lack a substantial amount of nutrients, specifically carbohydrates and dietary fiber, as well as micronutrients such as calcium, magnesium, potassium and vitamins A, B and B6.^154,155^ According to a recent research, KD delayed tumor growth but meanwhile accelerated cachexia onset, therefore shortening survival in a mouse model of IL-6-producing cancer. Excitingly, the same research group found that applying dexamethasone during KD treatment might delay cachexia onset without affecting the inhibition of tumor growth, providing fundamental insight into reversing the limitations of the clinical application of KD.^156^

### Protein restriction diet

The prevailing notion suggests that high protein intake, particularly among individuals under the age of 65, potentially escalates the risk of overall and cancer-related mortality.^157^ To establish a protein restriction diet, either dietary protein intake or the number of amino acids can be reduced.^140^ Recent research has demonstrated that dietary protein restriction is linked with a reduced incidence of tumor occurrence and a decreased risk of mortality.^158^

Dietary restriction of protein and certain amino acids, including serine, methionine, and branched-chain amino acids (BCAAs) such as leucine, isoleucine, and valine, has been shown to impede tumor growth.^159^ One mechanism through which protein restriction may inhibit tumor growth is via the IGF-1 signaling pathway. In melanoma and breast cancer mouse models, it has been observed that mice fed a low-protein diet (4% kcal protein) exhibit reduced IGF-1 levels and slower tumor progression compared to those fed a high-protein diet (18% kcal protein). A low-protein diet has been associated with reduced IGF-1 levels in patients aged 50–65 years, subsequently decreasing their risk of death from cancer. Conversely, a low-protein diet has been linked with an increased mortality rate in older patients (aged 65 and above), suggesting that a life-stage-specific approach to protein intake could optimize healthspan and longevity.^157^ Other potential mechanisms for cancer prevention that are mediated by protein restriction could involve mTOR signaling, amino acid metabolic programming, FGF21, and autophagy.^158^ In addition to these general effects, specific dietary restrictions on certain amino acids, such as serine and glycine, have been associated with prolonged survival in mouse models of various tumor types. The mechanisms underlying this observed survival benefit could include the correction of abnormal cellular nucleotide, protein, and lipid synthesis; improved mitochondrial function; and changes in epigenetic modifications.^160,161^

The antitumoral effect of a low-protein diet also hinges on promoting immunosurveillance against cancer, while the dietary restriction of amino acids may adversely affect the metabolic reprogramming of the TME in various ways. In multiple mouse models, reducing dietary methionine inhibited tumor growth and boosted antitumor immunity by increasing the quantity and cytotoxicity of tumor-infiltrating CD8^+^ T cells.^162^ Moreover, restricted intake of dietary protein or methionine/cystine has been shown to modify the infiltration and tumoricidal capacity of TAMs, leading to a significant increase in tumor-infiltrating CD8^+^ T cells and a decrease in the number of infiltrating MDSCs. Mechanistically, a protein-restricted diet inhibited mTOR pathway activation and increased macrophage acquisition of an antitumor phenotype by increasing the number of macrophages undergoing polarization to the M1 type.^163^ Macrophages might sense diet-derived cytosolic amino acids via the GTPase Rag, which subsequently regulates the expression of TFEB, TFE3 and mTORC1 when activated.^164^ Furthermore, an isocaloric diet that moderately reduced protein intake (by 25%) was shown to trigger an unfolded protein response (UPR) that depended on IRE1α in cancer cells. The increase in UPR activation, in turn, led to an increase in the recruitment of CD8^+^ T cells and enhanced antitumor immunosurveillance. Notably, intake of a low-carbohydrate diet did not exert the same effect.^165^ Although a low-protein isocaloric diet has been proven to reduce the concentration of amino acids in tumor tissues, it remains uncertain whether this reduction is limited to certain amino acids. Thus, further research is needed to explore the correlation between a low-protein isocaloric diet and the decrease in the levels of specific amino acids in tumors.

Interestingly, several studies have shown that high-protein diets may also benefit the restriction of tumor growth or clinical outcoming of cancer patients, which seem contradictory to the findings of the protein restriction diet discussed above. However, the underlying mechanisms are totally different. A high-protein diet increased the production of urinary urea in a tumor protein 53 (TP53)-mutated orthotopic bladder tumor mouse model, leading to the cascade modulation of ammonia in tumor cells, which induces tumor apoptosis.^166^ These findings challenge the former hypothesis that high urinary urea concentrations caused by a high-protein diet might serve as a potential carcinogenic factor in the bladder, suggesting the urgent need for further investigation.^167^ Applying a high-protein diet may improve the overall survival of older outpatients with advanced gastrointestinal cancer, which may improve the nutritional state of these patients with poor digestive system function.^168^

Moreover, there have been efforts to develop a series of drugs that mimic amino acid restriction. One focus of researchers in the cancer therapy field has been on glutamine metabolism, as cancer cells rely heavily on glutamine. Glutaminase inhibitors, for instance, have been shown to decrease tumor burden.^169,170^ The use of 6-diazo-5-L-oxo-norleucine (DON) promoted antitumor immunity by greatly favoring OXPHOS over glycolysis in CD8^+^ T cells while disrupting the metabolism of cancer cells.^171^ Notably, DON showed the ability to significantly inhibit the generation and recruitment of MDSCs and to reprogram M2-like TAMs into proinflammatory TAMs, which increased tumor antigen cross-presentation to T cells and enhanced the efficacy of immune checkpoint blockade (ICB).^172^ In addition, CB-839, which is considered the most effective glutaminase inhibitor, can be utilized alone or in combination with PD-1 inhibitors to treat solid or hematological malignancies.^173–175^ As previously mentioned, IDO and TDO are tryptophan catabolism enzymes, and inhibitors of these enzymes have been developed and evaluated in various clinical trials.^176^ For example, epacadostat is a novel compound that serves as an IDO1 inhibitor, suppressing systemic tryptophan catabolism.^177^ Both in vitro and in vivo studies have demonstrated that epacadostat can reduce tumor growth and promote the proliferation of T cells and NK cells.^178^ Furthermore, cyst(e)inase, a glutathione inhibitor that degrades cysteine and cystine, reduces tumor progression by elevating ROS levels and inducing tumor cell-selective ferroptosis.^179,180^

### High-salt diet

HSD has long been considered as a risk factor and trigger of malignancies. However, recent studies have provided new insights into the effect of sodium intake. As research continues, it is becoming increasingly clear that salt can accumulate in the interstitium and modulate immune cell differentiation, activation, and function through the effects of extracellular hypersalinity.^181^ In addition, consumption of a HSD can lead to elevated tissue sodium concentrations and affect immune responses within microenvironments, ultimately impacting the development of immune-regulated diseases such as infections and cancer.^182^

HSD, comprising 4% sodium chloride (NaCl), is recognized as a robust immunomodulator that is capable of eliciting a substantial inflammatory response.^183^ Indeed, research has shown that high salt conditions can inhibit tumor growth by enhancing antitumor immunity, particularly through the modulation of MDSC functions.^184^ According to a recent study, an HSD reduced the production of cytokines essential for the expansion of MDSCs and thus attenuated the accumulation of MDSCs within the tumor niche. As a result, the two primary types of MDSCs acquired different phenotypes: M-MDSCs differentiated into antitumor macrophages, and PMN-MDSCs adopted a proinflammatory phenotype, which led to the reactivation of T-cell antitumor functions.^185^ Furthermore, a high salt level has been found to induce the transformation of anti-inflammatory Tregs into proinflammatory Th1 cells, which led to the secretion of the inflammatory cytokine IFNγ.^186^ In another study, salt functioned as an adjuvant that enhanced the effectiveness of anti-PD-1 immunotherapy in tumor regression. Specifically, an HSD induces NK cell-mediated tumor immunity by suppressing PD-1 expression while increasing IFNγ levels and the serum hippurate concentration. Notably, hippurate is a microbial benzoate metabolism product that has been identified as a metabolic marker of effective PD-1 immunotherapy in responsive patients.^183^ Although the major antitumoural effect of HSD is modulating immune cell function, mechanisms other than immunomodulation have also been discovered. For instance, HSD suppressed tumor growth and lung metastasis in a murine model of breast cancer, possibly by inducing hyperosmotic stress or through mimicking CR.^187^

Nevertheless, despite the potential benefits of salt intake on cancer treatment effectiveness, high salt intake can also lead to the development of a proinflammatory state, which can negatively impact cancer outcomes.^188^ High salt intake is a risk factor for various types of cancer in humans, including lung, testicular, bladder, renal cell, pancreatic, esophageal, and gastric cancer.^182^ HSD has been shown to induce chronic inflammation, which may in turn incite continuous cell proliferation, DNA damage, or cancer transformation. However, whether there is a connection remains uncertain.^188^ IL-17, specifically IL-17A, plays an important role in the mechanism of action of HSD. Evidence suggests that high salt intake can induce the differentiation of Th17 cells, a prominent source of IL-17A.^189^ The overproduction of IL-17A can lead to inflammation and other immune responses that contribute to various pathologies. Furthermore, in the case of breast cancer, an HSD has been found to promote tumor progression and lung metastasis, increase the proportion of Th17 cells, and activate the MAPK/ERK signaling pathway in breast cancer cells through the secretion of IL-17F. The increase in the secreted IL-17F level results in the unregulated expression of protumor genes and the induced inflammatory responses, ultimately accelerating the proliferation, migration and invasion of breast tumors.^190^ In addition, the combination of high NaCl concentrations with subeffective IL-17 has been proven to reduce reactive nitrogen and oxygen species (RNS/ROS) levels and enhance the growth of breast cancer cells.^191,192^ Recent research has also demonstrated that intake of an HSD can disrupt the development and function of NK cells in mice.^193^ Therefore, it can be concluded that dietary salt may exert dual effects on tumorigenesis, and the contradictory results obtained may be due to variations in the effects of high salt concentrations on tumors in different tissues and during different phases of tumor development.

### Obesity and high-fat diet

Obesity, a serious health issue characterized by excessive body fat, is a known risk factor for multiple types of cancer. It can be induced or exacerbated by HFD, characterized by the consumption of foods rich in saturated fats and cholesterol.^194^ Obesity can induce systemic metabolic disruptions within the body, leading to dyslipidemia, hypercholesterolemia, insulin resistance, alterations in hormone levels, and changes in the baseline inflammation status.^195^ Conversely, a low-fat diet, typically associated with reduced total fat intake, can potentially lower the risk of certain types of cancer.^196,197^ Given that both HFD and obesity are major factors influencing cancer risk, the forthcoming discussion will primarily focus on these aspects. By diving deeper into the mechanisms by which HFD and obesity affect cancer development and progression, we aim to provide a more comprehensive understanding of this intricate relationship.

Dietary obesity is associated with multiple factors related to cancer occurrence and exacerbation of immune suppression in tumor niches.^198^ In the context of obesity, increased hepatic expression of the unconventional prefoldin RPB5 interactor (URI) has been shown to couple nutrient surplus with inflammation, leading to nonalcoholic steatohepatitis (NASH) and consequent HCC. This process involves URI-induced DNA damage in hepatocytes triggering Th17 lymphocyte-mediated inflammation, and subsequent IL-17A-induced adipose tissue neutrophil infiltration, which promotes insulin resistance and hepatic fat accumulation, thereby inducing NASH and HCC.^199^ Notably, obesity also accelerates *Helicobacter felis*-induced gastric carcinogenesis by enhancing the trafficking of immature myeloid cells and the Th17 response. This exacerbates proinflammatory immune responses, characterized by cross-talk between inflamed gastric and adipose tissues, thereby contributing to a protumorigenic gastric microenvironment.^200^

Diet-induced obesity has been shown to elevate nitric oxide (NO) production, which enhances tumor growth. This is primarily due to the recruitment of macrophages and the overexpression of inducible NO synthase as a result of HFD.^201^ Additionally, in response to HFD intake, IL-6-mediated inflammation has been shown to accelerate prostate cancer tumor growth and increase the fraction of MDSCs and the M2/M1 macrophage ratio.^202^ The effects of diet-induced obesity extend to the microenvironment of colitis-associated CRC. Here, diet-induced obesity has been shown to increase IL-6 expression and promote the polarization of macrophages into M2-like macrophages, enhancing the production of CC-chemokine-ligand (CCL) 20. CCL20 recruits CC-chemokine receptor 6 (CCR6)-expressing B cells and γδ T cells, ultimately leading to colitis-associated CRC progression.^203^ In animal models of HFD-induced obesity, the infiltration rate of TAMs and the expression of cytokines in M2-like macrophages were increased, enhancing tumor growth and metastasis. However, ablation of VEGFR-1 signaling can reverse the abnormal TME associated with obesity and reprogram TAMs to promote their acquisition of the M1 phenotype.^204^

The intake of an HFD has been shown to significantly increase the incidence of oral squamous cell carcinoma (OSCC) by expanding MDSCs within the local immune microenvironment.^205^ Obesity induced by diet can also trigger the accumulation of PMN-MDSCs, leading to Fas/FasL-mediated apoptosis of tumor-infiltrating CD8^+^ T cells and causing resistance to immunotherapy in breast cancer treatment.^206^ Obesity has been shown to suppress the infiltration and function of CD8^+^ T cells, which was linked to decreased chemokine production, reduced fatty acid availability, and alterations in amino acid metabolism.^207,208^ Moreover, based on findings from mouse models, obesity reduced the number and function of CD4^+^ T cells in the TME of CRC, leading to a compromised antitumor response of both CD4^+^ and CD8^+^ T cells and ultimately accelerating disease progression.^209^ Furthermore, considerable evidence shows that obesity-associated adipocytes in pancreatic ductal adenocarcinoma can secrete IL-1β to attract tumor-associated neutrophils (TANs), which subsequently activate pancreatic stellate cells and contribute to tumor growth.^210^

HFD or diet-induced obesity may induce tumor metastasis. HFD has been proven to increase palmitate secretion from alveolar type 2 cells and nuclear factor-kappaB subunit p65 acetylation in the lung to prepare a premetastatic niche.^211^ HFD-induced fatty liver may promote liver metastasis by facilitating the secretion of hepatocyte-derived extracellular vesicles (EVs), which transfer Yes-associated protein (YAP) signaling-regulating microRNAs, hence elevating nuclear YAP expression, CYR61 expression, and M2-like macrophage infiltration.^212^ Another mechanism of HFD-induced liver metastasis is the upregulation of NOD-like receptor C4 (NLRC4), which further induces M2-like macrophage activation and IL-1β processing. An alteration from an indolent to a metastatic state may be stimulated by HFD-induced lipid accumulation in prostate tumors, the mechanism of which may be related to the sterol regulatory element-binding protein (SREBP)-related prometastatic lipogenic program.^213^ In addition, it is widely acknowledged that the fatty acid receptor CD36 plays an important role in HFD-related metastasis promotion by enhancing the metastatic potential of CD36^+^ metastasis-initiating cells.^214^ However, a recent study revealed that CD36 may prevent palmitate-induced lipotoxicity rather than facilitating HFD-driven metastasis, suggesting that further investigations of the dual effects of CD36 are needed.^215^

An elevated cholesterol level is an obesity comorbidity, and studies suggest that the effects of obesity on cancer may be partly mediated by increased cholesterol levels.^216^ In fact, a high-cholesterol diet (HCD) alone has been shown to promote macrophage infiltration and significantly enhance the growth of CRC tumors.^217^ One mechanism by which HCD promotes CRC progression is through the inhibition of the CD8^+^ T-cell response. Specifically, macrophages with infiltration driven by HCD can secrete CCL5, which obstructs the activation of CD8^+^ T cells, thereby facilitating the evasion of immune system surveillance by CRC cells.^218^ 27-Hydroxycholesterol (27-HC) is a crucial mediator of the effects of dietary cholesterol on cancer metastasis. This oxysterol is synthesized through the action of the CYP27A1 enzyme and is present at high levels in the circulatory system.^219^ Oxysterol has been shown to modulate the TME by recruiting immunosuppressive neutrophils to the metastatic niche, facilitating cancer progression.^220^ However, some studies have reported conflicting findings regarding the effects of high serum cholesterol levels on cancer progression. For instance, one study showed that high serum levels of cholesterol attributed to HCD intake increased the accumulation of NK cells and promoted their effector functions to reduce the growth of liver tumors in mice.^221^ However, further studies are needed to understand these conflicting findings.

In expanding on the relationship between HFD and tumor promotion, it is worth noting that the tumor-promoting effect of HFD is not universal and depends largely on the subtype of fatty acids involved. Mouse models of breast cancer developed comparable obesity levels from an HFD of either cocoa butter or fish oil. However, the consumption of the cocoa butter HFD, which is high in saturated fatty acids, led to faster mammary tumor growth and increased protumor macrophages and IL-10 expression while reducing B-cell and CD8^+^ T-cell infiltration. On the other hand, the fish oil HFD, which is rich in omega-3 fatty acids, disrupted the typical obesity-tumor growth link and reduced the number of protumor macrophages.^222^ This effect of dietary omega-3 fatty acids is mediated by host GPR120 and has also been shown to inhibit prostate cancer.^223^ Moreover, oleic acid (OA) and linoleic acid (LA) are the most common unsaturated fatty acids in dietary oils. While both an HFD rich in OA and an HFD rich in LA can similarly induce obesity in mice, a diet high in LA specifically encourages the growth of mammary tumors. Furthermore, an LA-rich HFD can impair antitumor T-cell responses via the induction of mitochondrial dysfunction.^224^ Based on these findings, it appears that modulating dietary oil composition may constitute a promising strategy for enhancing immune function in both the prevention and treatment of obesity-associated cancers. By carefully selecting and balancing the types of fatty acids in HFDs, it may be possible to reduce the tumor-promoting effects of obesity while simultaneously increasing immune responses against tumors. Further research in this area may help to identify more precise dietary interventions that can ultimately improve outcomes for individuals at risk of developing obesity-associated cancers.

## Potential role of dietary factors in cancer treatment

### Immunotherapy

Recent studies have highlighted the pivotal influence of the TME on the efficacy of immunotherapy in cancer treatment.^225^ Immunotherapy, recognized as a substantial advance in cancer treatment, has revolutionized the field of oncology by augmenting the body’s innate defenses to effectively target and eliminate malignant cells.^226^ Various forms of cancer immunotherapy have been developed, including oncolytic virus therapies, cancer vaccines, cytokine therapies, adoptive cell transfer, and ICIs, all of which have shown promise in clinical practice.^227^ Among these therapies, ICIs are perhaps the most important, as they are antibody-based drugs that can eliminate the influence of tumor-specific CD8^+^T cells.^228^ In particular, ICIs targeting PD-1 or its ligand PD-L1 have demonstrated notable clinical efficacy in the treatment of various advanced cancers.^229^

Extensive research has been conducted to identify the effects of various dietary substances and patterns on tumor growth, metastasis and TME reprogramming, which has led to the consideration of nutritional intervention as a possible strategy for increasing the efficacy of tumor treatment^230,231^ (Tables 2, 3). The decline in T-cell functionality with aging, a widely documented phenomenon, is linked to a reduced efficacy of anti-OX40 immunotherapy in murine models.^232^ CR not only preserves T-cell function but also improves the response of aged CD4^+^ T-cell populations to anti-OX40 therapy.^233^ When used in combination with immunogenic cell death (ICD)-inducing chemotherapy and immunotherapy, CRMs potentially enhance the efficacy of cancer treatments through synergistic effects.^234^ Preclinical studies have shown that STF, which serves as an adjunct to various cancer treatments, may bolster antitumor immunity by attenuating immunosuppressive conditions and amplifying CD8^+^ T-cell cytotoxicity.^235^ For example, an experimental study of non-small cell lung cancer demonstrated that STF sensitized cancer cells to anti-PD-1 therapy. The antitumor efficacy of combination therapy was achieved by inhibiting IGF-1-IGF-1R signaling in cancer cells, boosting the intratumoral CD8 cell: Treg ratio in the TME.^132^ Furthermore, intake of an FMD has been shown to enhance the effectiveness of immunotherapy against triple-negative breast cancer with low immunogenicity by affecting the TME. Specifically, intake of an FMD has been shown to reactivate T_eff_ cells that underwent early exhaustion, shift cancer metabolism from glycolytic to OXPHOS, and reduce the collagen deposition rate.^236^ These effects led to the increased efficacy of anti-PD-L1 and anti-OX40 immunotherapy. These results suggest that combining immunotherapy with dietary restriction may lead to profound synergistic effects.

**Table 2:** Preclinical studies showing effects of dietary intervention on cancer therapy

Dietary intervention	Cancer type	Comparison of treatment	Results	References
Calorie restriction	Breast cancer	Radiotherapy	T_eff_/Tregs ratio↑, PD-1^+^CD8^+^ T cells↑ Tregs↓	^261^
CRM hydroxycitrate	Fibrosarcomas Colorectal cancer Lung cancer	Chemotherapy	Autophagy in tumor cells↑ Tregs↓	^122^
Fasting or fasting-mimicking diet	Lung cancer	Immunotherapy	CD8/Treg ratio↑, NK cells↑ Tregs↓, CD19^+^ B cells↓, PD-1^+^CD8^+^ T cells↓, PD-1^+^CD4^+^ T cells↓	^132^
	Pancreatic cancer	Chemotherapy	Levels of equilibrative nucleoside transporter (hENT1) in tumor cells↑ Levels of ribonucleotide reductase M1 (RRM1) in tumor cells↓	^251^
	Breast cancer	Chemotherapy	ROS in tumor cells↑	^252^
		Immunotherapy	T cells↑, CD8^+^ T cells↑, GZMB^+^CD8^+^ T cell↑, Ki67^+^CD8^+^ T cells↑, γδ T cells↑, GZMB^+^ γδ T cells↑, Ki67^+^ γδ T cells↑, Ki67^+^FOXP3^-^CD4^+^ T cells↑, OX40^+^FOXP3^-^CD4^+^ T cells↑, PD-1^+^FOXP3^-^CD4^+^ T cells↑, Ki67^+^ Tregs↑, OX40^+^ Tregs↑, PD-L1^+^ PMN-MDSCs↑, macrophages↑, PD-L1^+^ macrophages↑, Tox^int^CD8^+^ T cells↑, Tox^int^PD-1^int^CD39^low^CD8^+^ T cells↑ PMN-MDSCs↓, M2-like macrophages↓	^236^
		Endocrine therapy	Circulating IGF1, insulin, and leptin levels↓, AKT-mTOR signaling↓	^265^
	Breast cancer Melanoma	Chemotherapy	CD8^+^ T cells↑, CD3^+^ T cells↑, granzyme-B↑ HO-1↓, Tregs↓	^136^
	Lung cancer Breast cancer Colorectal cancer	TKIs	E2F-dependent transcription inhibition↑ MAPK signaling pathway↓	^266^
	Hepatocellular carcinoma	TKIs	Glucose↓, AKT/mTOR signaling↓	^267^
Fasting-mimicking diet+ vitamin C	Colorectal cancer	Chemotherapy	Reactive iron and oxygen species in tumor cells↑	^253^
Fasting-mimicking diet+ ferroptosis inducer	Colorectal cancer	Chemotherapy	Autophagy in tumor cells↑	^254^
Ketogenic diet	Colorectal cancer	Immunotherapy	IFNγ^+^CD8^+^ T cells↑, TNFα^+^CD8^+^ T cells↑	^238^
	Neuroblastoma	Chemotherapy	Tumor burden↓	^258^
		Chemotherapy	Serine, glutamine and glycine↑ Tumor blood-vessel density and intratumoral hemorrhage↓, serum levels of essential amino acids↓	^259^
	Pancreatic cancer	Chemotherapy	Tumor NADH levels↑	^260^
		Radiotherapy	Oxidative stress in tumor cells↑	^263^
		PI3K inhibitors	Hyperglycemia↓, insulin secretion↓, mTORC1 signaling↓	^268^
	Lung cancer	Radiation or radio-chemotherapy	Oxidative stress in tumor cells↑	^262^
Dietary restriction of protein/80% methionine-restricted diet	Prostate cancer	Immunotherapy	CD8^+^ T cells↑, CD8^+^ T cells/M1-like ratio↑, CD8^+^ T cells/M2-like ratio↑, CD8^+^ T cells/M-MDSC ratio↑, CD8^+^ T cells/PMN-MDSC ratio↑ M2-like TAMs↓	^163^
Dietary methionine restriction	Colorectal cancer	Immunotherapy	CD8^+^ T cells↑	^162^
	Colorectal cancer Soft-tissue sarcoma	Radiotherapy Antimetabolite chemotherapy.	Alterations in one-carbon metabolism	^264^
Dietary serine deprivation	Lung cancer Colorectal cancer	Biguanide treatment	Serine↓, glycolysis↓	^269^
High-salt diet	Breast cancer Melanoma	Immunotherapy	CD4^+^ T cells↑, CD8^+^ T cells↑	^185^
	Melanoma	Immunotherapy	PD-1^+^ NK cells↓	^183^
High-fat diet	Breast cancer	Immunotherapy	PMN-MDSCs↑, the expression of *Cxcl1*↑	^206^
	Melanoma	Immunotherapy	CD3^+^ T cells↑, CD8^+^ T cells↑, CD8^+^/CD4^+^ ratio↑	^246^
	Ovarian cancer	Chemotherapy	Fibrosis↑, M1-like macrophages↓, M2-like macrophages↓, M2/M1 macrophage ratio↑	^442^
Inulin gel	Colorectal cancer	Immunotherapy	CD8^+^ cells↑, AH1 tetramer^+^CD8^+^ T cells↑, DCs↑, CD4^+^ T cells↑, Tcf1^+^PD-1^+^CD8^+^ T cells↑, neutrophils↑, M1/M2 macrophages ratio↑ PD-1^+^CD8^+^ T cells↓, M2-like macrophages↓, Tregs↓	^303^
Pectin	Colorectal cancer	Immunotherapy	CD4^+^ T cells↑, CD8^+^ T cells↑, IFNγ^+^CD8^+^ T cells↑	^304^

**Table 3:** Clinical trials of dietary intervention combined with cancer treatment

Dietary intervention	NCT	Therapeutic intervention	Disease	Status
Calorie restriction	NCT01819233	Surgery and radiotherapy	Stage 0-I breast cancer	Completed
	NCT02792270	Pre-operative radiotherapy	Sarcoma	Unknown
	NCT01802346	Chemotherapy	Breast or prostate cancer	Recruiting
Cyclic, 5-day calorie restriction	NCT05703997	Atezolizumab	Small cell lung cancer	Not yet recruiting
Calorie restriction and exercise	NCT03131024	Anthracycline-containing chemotherapy	Breast cancer	Completed
Intermittent calorie restriction and plant-based diet	NCT05359848	Chemotherapy	Cancer	Recruiting
Low-carbohydrate diet	NCT02149459	Radiotherapy with or without metformin	Recurrent brain cancer	Unknown
Very low carbohydrate diet	NCT04035096	High dose intravenous vitamin C	Stage IV colon cancer with KRAS and BRAF mutation	Unknown
Intermittent fasting	NCT01175837	Chemotherapy	Cancer	Completed
	NCT02607826	Chemotherapy	Solid tumors	Unknown
	NCT06015087	Chemotherapy	Breast cancer	Recruiting
	NCT01304251	Chemotherapy	Breast cancer	Completed
	NCT05722288	Radiotherapy and/or chemotherapy and/or hormone therapy	Prostate, cervical or rectal cancer	Recruiting
	NCT04247464	Chemotherapy	Colorectal cancer	Enrolling by invitation
Nightly fasting	NCT05023967	Chemotherapy and metformin	Early breast cancer	Recruiting
Prolonged nightly fasting	NCT05083416	Immunotherapy	Advanced head & neck cancer	Active, not recruiting
Alternate day fasting	NCT05990426	Chemotherapy	Endometrial, ovarian, fallopian tube or primary peritoneal cancer	Not yet recruiting
5:2 intermittent fasting	NCT05861362	Radiotherapy	Breast Cancer	Completed
Time restricted eating with or without Mediterranian diet	NCT05259410	Chemotherapy	Breast Cancer	Recruiting
Intermittent fasting or vegan diet	NCT03162289	Chemotherapy	Gynecological cancer	Active, not recruiting
Intermittent fasting or Mediterranian diet	NCT02710721	Chemotherapy with or without hormone therapy	Advanced metastatic prostate cancer	Completed
Fasting-mimicking diet	NCT03595540	Active cancer treatment	Cancer	Completed
	NCT03709147	Metformin hydrochloride, cisplatin, carboplatin, pemetrexed, pembrolizumab	Advanced LKB1-inactive lung adenocarcinoma	Unknown
	NCT05763992	Chemoimmunotherapy	Triple-negative breast cancer	Recruiting
	NCT05503108	Neoadjuvant chemotherapy (ddAC, T)	HR+, HER2- breast cancer	Recruiting
	NCT02126449	Neoadjuvant chemotherapy (AC>T)	HER2- breast cancer	Completed
	NCT04248998	Neoadjuvant chemotherapy (AC>T) with or without metformin	Triple-negative breast cancer	Active, not recruiting
	NCT03340935	Standard cancer treatment	Malignancies with the exception of small cell neuroendocrine tumors	Completed
	NCT05921149	Carboplatin and paclitaxel	Advanced or recurrent ovarian, fallopian tube and primary peritoneal cancer	Not yet recruiting
Ketogenic diet	NCT05119010	Nivolumab and ipilimumab	Metastatic renal cell carcinoma	Recruiting
	NCT05938322	Neoadjuvant radiotherapy	Locally advanced nonmetastatic rectal adenocarcinoma	Not yet recruiting
	NCT04316520	Nivolumab + ipilimumab, pembrolizumab + axitinib, sunitinib or pazopanib	Metastatic renal cell carcinoma	Recruiting
	NCT03962647	Letrozole	Early-stage ER+, HER2- breast cancer	Active, not recruiting
	NCT03535701	Paclitaxel	Stage IV breast cancer	Completed
	NCT05234502	Neoadjuvant chemotherapy (AC>T)	Breast cancer	Not yet recruiting
	NCT05708352	Chemotherapy and/or radiotherapy	Glioblastoma	Recruiting
	NCT03451799	Radiotherapy and temozolomide	Glioblastoma	Active, not recruiting
	NCT02302235	Radiotherapy and temozolomide	Glioblastoma multiforme	Completed
	NCT02939378	Chemotherapy	Recurrent glioblastoma multiforme	Unknown
	NCT04631445	Nab-paclitaxel, gemcitabine, and cisplatin	Metastatic pancreatic ductal adenocarcinoma	Recruiting
	NCT02516501	Radio(chemo)therapy	Breast, head & neck or colorectal carcinoma	Completed
	NCT02983942	Chemotherapy (HD-MTX)	Primary central nervous system lymphoma	Unknown
Energy restricted ketogenic diet	NCT01535911	Chemotherapy and radiotherapy	Glioblastoma multiforme	Active, not recruiting
Intermittent fasting and time-restricted modified ketogenic diet between fasts	NCT04730869	Chemoradiotherapy and adjuvant chemotherapy	Glioblastoma multiforme	Recruiting
Modified ketogenic diet or medium chain triglyceride ketogenic diet	NCT03075514	Chemotherapy, radiotherapy or chemoradiotherapy	Glioblastoma	Completed
Modified Atkins diet	NCT02768389	Bevacizumab	Recurrent glioblastoma or other grade IV malignant glioma	Completed
	NCT03278249	Temodar and radiotherapy	Primary malignant glioma	Active, not recruiting
Low protein diet	NCT03329742	Sipuleucel-T	Metastatic castrate-resistant prostate cancer	Completed
	NCT05356182	Immunotherapy	Solid tumors	Recruiting
High protein diet	NCT05677958	Chemo(radio)therapy or immunotherapy	Colorectal or non-small cell lung cancer	Completed
	NCT03559881	Chemotherapy and/or immunotherapy	Non-small cell lung cancer	Completed
Supplementation of poly-unsaturated n-3 fatty acids and high-protein diet	NCT04965129	Immunotherapy, chemotherapy and tyrosine kinase Inhibitors	Non-small cell lung cancer	Recruiting
Mediterranean diet	NCT04045392	Adjuvant hormone therapy	Breast cancer	Unknown
	NCT04534738	Chemotherapy	Cancer	Completed
Mediterranean diet with or without exercise	NCT05839210	Chemotherapy	Lymphoma	Recruiting
Plant-based Mediterranean diet (olive oil supplementary)	NCT01083771	Androgen deprivation therapy	Prostate cancer	Completed
Mediterranean-DASH intervention for neurodegenerative delay	NCT05984888	Chemotherapy, targeted therapies or endocrine therapy	HR+ breast cancer	Recruiting
High-fiber diet	NCT05805319	Immune checkpoint inhibition	Non-small cell lung cancer	Recruiting
	NCT04534075	Radiotherapy	Gynecological, colorectal, anal, prostate, or urinary bladder cancer	Recruiting
	NCT01549782	Radiotherapy	Gynecological cancer	Completed
	NCT00888147	Radiotherapy	Head & neck cancer	Completed
Low-fiber diet or high-fiber diet	NCT01170299	Radiotherapy	Gynecological, urological (bladder), colorectal or anal cancer	Completed
Isocaloric high-fiber diet	NCT04645680	Pembrolizumab or nivolumab	Unresectable melanoma	Recruiting
High-fiber, plant-based diet and exercise	NCT04866810	ipilimumab + nivolumab, relatlimab + nivolumab, pembrolizumab or nivolumab	Melanoma	Recruiting
Calorie-restricted plant-based diet and exercise	NCT04298086	Anastrozole, letrozole or exemestane	HR+ breast cancer	Active, not recruiting
Low fat diet	NCT00002564	Adjuvant therapy with or without either chemotherapy or endocrine therapy	Early-stage breast cancer	Completed
Carbohydrate-restricted, high-fat diet	NCT04253808	Radiotherapy	Head & neck cancer	Completed
Oral vitamins A and E	NCT00228319	Chemotherapy	Ovarian cancer	Completed
Oral vitamin A and folic acid	NCT05720559	Oxaliplatin, cetuximab and metronidazole	Prehepatic CTC+ colorectal cancer	Not yet recruiting
	NCT05774964	Oxaliplatin, cetuximab and metronidazole	Colorectal cancer with liver metastases	Not yet recruiting
Vitamins B6 and B12 supplementation	NCT00659269	Chemotherapy	Cancer	Completed
Vitamin B12 and folic acid supplementation	NCT02679443	Chemotherapy	Non-small cell lung cancer	Completed
	NCT00609518	Pemetrexed and dexamethasone	Non-small cell lung cancer	Completed
	NCT00216099	Pemetrexed	Hormone refractory prostate cancer	Completed
Oral vitamin D	NCT03467789	Radiotherapy	Basal cell carcinoma	Recruiting
	NCT04864431	Chemotherapy	Epithelial ovarian cancer	Recruiting
	NCT02603757	Chemotherapy	Colorectal cancer	Completed
	NCT04091178	Chemotherapy	Breast cancer	Completed
	NCT04677816	Chemotherapy	Triple-negative breast cancer	Recruiting
	NCT03331562	Pembrolizumab	Metastatic pancreatic ductal adenocarcinoma	Completed
Oral vitamin D and Omega-3	NCT05331807	Chemotherapy	Breast cancer	Recruiting
Oral vitamin E	NCT03613389	Chemotherapy	Pediatric cancer	Unknown
	NCT00363129	Chemotherapy	Cancer	Completed
Oral vitamin E and Hydrogen-rich water	NCT04713332	Radiotherapy	Rectal cancer	Unknown
Antioxidant-deficient diet	NCT00486304	Chemotherapy and radiotherapy	Oropharyngeal cancer	Completed
Anti-inflammatory diet	NCT03994055	Chemo-radiotherapy and brachytherapy	Cervical cancer	Active, not recruiting
Low copper diet	NCT00003751	Radiotherapy and penicillamine	Glioblastoma multiforme	Completed
Paleolithic diet and exercise	NCT04574323	Radiotherapy	Breast cancer	Completed
Low-residue diet	NCT00258401	Radiotherapy	Uterine, cervical or prostate cancer	Completed

KD also enhances the antitumor effects of PD-1 blockade alone or in combination with anti-CTLA-4 antibodies. Mechanistically, the principal ketone body 3-hydroxybutyrate (3HB) in a KD prevented the ICB-mediated upregulation of PD-L1 on myeloid cells while simultaneously promoting the expansion of CXCR3^+^ T cells.^237^ Similarly, KD enhanced the effectiveness of anti-CTLA-4 immunotherapy by reducing PD-L1 protein levels and augmenting the expression of interferons and antigen presentation-related genes. When combined with immunotherapy, the intake of a KD can reshape the TME by increasing the population of CD8^+^ TILs, macrophages and CD86^+^ DCs. Mechanistically, the activation of AMPK via KD intake is the key molecular event that promotes immunotherapy efficacy. This activated AMPK phosphorylates PD-L1 on Ser283, which interrupts its association with CMTM4 and results in PD-L1 degradation. Furthermore, AMPK phosphorylates EZH2, which impedes polycomb repressive complex 2 (PRC2), leading to an increase in interferons and antigen-presenting gene expression.^238^

Combining a protein-restricted diet with a vaccine or anti-PD-1 therapy has been shown to significantly inhibit tumor growth and prolong survival.^239^ Notably, treatment with a methionine-/cystine-restricted diet significantly increased the number of tumor-infiltrating CD8^+^ T cells and cytotoxic granzyme B^+^CD8^+^ T cells, which was further enhanced when combined with immunotherapy.^163^ Another study confirmed the inhibitory effect of dietary methionine restriction on tumor growth and its ability to synergize with PD-1 blockers to increase tumor control. Mechanistically, this dietary approach reduced the number of metabolites, such as S-adenosylmethionine (SAM), which controls N6-methyladenosine (m6A) methylation reactions, in cancer cells. A reduction in the SAM level altered the m6A modification rate and decreased the expression of PD-L1 and V-domain Ig suppressor of T-cell activation (VISTA) in cancer cells.^162^ Moreover, the enzyme cyst(e)inase breaks down cystine and cysteine, thereby bolstering T-cell-mediated antitumor immunity and inducing ferroptosis in tumor cells when combined with PD-L1 blockade.^240^ IDO1 is a critical enzyme in the tryptophan–kynurenine pathway and has been identified as a promising immunomodulatory target.^241^ A phase 1/2 (ECHO-202/KEYNOTE-037) trial evaluating the effectiveness of the IDO1 inhibitor epacadostat combined with pembrolizumab on advanced solid tumors showed a high objective response rate (ORR) of 40.3% overall and 61.9% in malignant melanoma patients, demonstrating promising antitumor efficacy.^242^ Unfortunately, phase 3 trials failed to confirm these benefits. The ECHO-301/KEYNOTE-252 trial showed that combining epacadostat with pembrolizumab failed to prolong progression-free survival (PFS) or overall survival (OS) compared to pembrolizumab alone in patients with advanced melanoma.^243^

Despite being linked to T-cell dysfunction and poor cancer prognosis, obesity has paradoxically been shown to enhance the response to anti-PD-1/PD-L1 immunotherapy.^244^ Recent research suggests that immunotherapy yielded superior outcomes in obese patients, evidenced by an improved response rate and extended PFS and OS, in comparison to lean patients.^245^ However, obesity also promoted tumor growth and T-cell exhaustion, leading to increased PD-1 expression and dysfunction, partly due to high leptin levels. Despite this outcome, PD-1-mediated T-cell dysfunction in individuals with obesity was found to significantly enhance tumor responsiveness to PD-1/PD-L1 inhibitors, as confirmed by preclinical and clinical data.^246^ Therefore, obesity seems to be a double-edged sword for cancer immunotherapy, and the underlying mechanisms remain unclear and require further investigation.

### Chemotherapy

Chemotherapy, a cornerstone of traditional cancer treatment, employs drugs to destroy rapidly dividing cells, a defining characteristic of cancer.^247^ Despite its widespread use and undeniable efficacy in many cases, chemotherapy often has substantial side effects due to its impact on healthy cells.^248^ Additionally, individual responses to chemotherapy can vary greatly and are influenced by a multitude of factors, including genetics, tumor characteristics, and, intriguingly, diet.^15^ A growing body of research now highlights the role of dietary interventions in modulating the effectiveness of chemotherapy, emphasizing the need to further understand these interactions for improved therapeutic outcomes.

Due to their expression of oncogenes, cancer cells are more susceptible to the effects of fasting and CR than are normal cells, an effect termed ‘differential stress resistance’.^14,249,250^ Based on this characteristic, CRM hydroxycitrate has been shown to increase sensitivity to chemotherapy by eliciting an adaptive cellular immune response, resulting in a decrease in the number of tumor-infiltrating Tregs into the tumor niche in various tumor models.^122^

Emerging research also suggests a profound influence of fasting or FMD on the efficacy of chemotherapy. In vitro studies indicate that fasting cycles not only retard tumor growth but also sensitize a wide array of cancer cell types to chemotherapy.^14^ This heightened sensitivity has been observed in various contexts, including the enhancement of gemcitabine efficacy in mice with prostate cancer xenografts and the increased efficacy of chemotherapy in triple-negative breast cancer via the upregulation of ROS.^251,252^ FMD combined with vitamin C can potentially increase the effectiveness of chemotherapy for treating KRAS-mutant cancer cells by reversing the vitamin C-induced upregulation of HO-1 and ferritin.^253^ Furthermore, when combined with a ferroptosis inducer, FMD can effectively eliminate slow-cycling, chemotherapy-resistant cells, suggesting a potential strategy for enhancing the sensitivity of certain difficult-to-treat cancers to chemotherapy through dietary interventions.^254^ Interestingly, fasting can also counteract certain adverse effects of chemotherapy. For instance, it has been demonstrated to enhance self-renewal in hematopoietic stem cells and mitigate the immunosuppression induced by cyclophosphamide chemotherapy in mice.^255^ In tumor-bearing mice, both prolonged fasting and FMDs can induce specific stress resistance responses, enhancing chemotoxicity in cancer cells while protecting normal cells.^256^ This dual action is partly mediated by the reduction in IGF-1 and glucose levels, thus shielding normal cells and organs from chemical toxicity.^250^ The potential of FMD in clinical settings has been supported by the ‘DIRECT’ study involving HER2-negative stage II/III breast cancer patients. This study revealed that treatment with FMD, administered three days prior to and during neoadjuvant chemotherapy, enhanced therapeutic efficacy without increasing toxicity or reducing chemotherapy-induced DNA damage in T cells.^257^ Collectively, these findings highlight the potential of fasting and FMD as adjuncts to chemotherapy, warranting further exploration and clinical testing.

In addition to slowing tumor growth, KD also sensitizes tumor cells to classic chemotherapy. For example, the combination of KD with metronomic cyclophosphamide significantly enhances antitumor effects, resulting in the regression of neuroblastoma tumors.^258,259^ Similarly, in pancreatic cancer, cotreatment with KD and cytotoxic chemotherapy substantially elevates tumor NADH levels, synergistically suppressing tumor growth and tripling survival benefits compared to chemotherapy alone.^260^

### Radiotherapy

Dietary interventions have emerged as promising strategies for enhancing the efficacy of radiotherapy in cancer treatment. For instance, CR combined with radiotherapy, has been shown to modulate the TME in a triple-negative breast cancer model by decreasing the number of intratumoral Tregs, increasing the CD8^+^ cell: Treg ratio, and upregulating PD-1 expression on CD8^+^ T cells. Furthermore, compared with patients who received radiotherapy alone, breast cancer patients who underwent CR concurrently with radiotherapy exhibited a significant reduction in the serum levels of immunosuppressive cytokines, suggesting potential benefits of CR in mitigating radiation-induced immunosuppression.^261^

When combined with radiation or radiochemotherapy, KD slows tumor growth in lung cancer xenografts, potentially through a mechanism involving increased oxidative stress.^262^ Additionally, KD was shown to enhance radiation sensitivity in a pancreatic cancer xenograft model, suggesting potential improvements in therapeutic outcomes. However, phase 1 clinical trials in patients with locally advanced non-small cell lung cancer and pancreatic cancer showed suboptimal compliance with the diet, indicating challenges in practical application.^263^

Moreover, other dietary restrictions, such as methionine deprivation, have shown promising results in enhancing the efficacy of radiation and antimetabolite chemotherapy. In patient-derived xenograft and autochthonous tumor mouse models, methionine restriction sensitized tumor cells to these treatments, possibly via alterations in one-carbon metabolism.^264^

### Other therapies

In hormone receptor-positive breast cancer mouse models, periodic fasting or an FMD can enhance the therapeutic effects of endocrine agents such as tamoxifen and fulvestrant. This enhancement is believed to occur through a reduction in circulating IGF1, insulin, and leptin levels and suppression of AKT-mTOR signaling. Concurrent administration of these dietary strategies with a therapeutic regimen of fulvestrant and palbociclib has been associated with prolonged tumor regression and reversal of treatment resistance. Analogous metabolic alterations found in patients on an FMD during estrogen therapy suggest the potential of diet as an adjuvant in treating hormone receptor-positive breast cancer.^265^

In addition to their effects on hormone-driven cancers, fasting or FMD has also been shown to enhance the efficacy of tyrosine kinase inhibitors (TKIs) across different cancer cell lines. Mechanistically, these effects are attributed to the increased ability of TKIs to block cancer cell growth and inhibit the MAPK signaling pathway under starvation conditions.^266^ Another study reported that in HCC cells, xenografts, and patient-derived organoids, fasting improved the therapeutic response to sorafenib through the regulation of glucose transporters and proapoptotic protein expression by p53.^267^

KD has also shown promise in supporting the effectiveness of phosphatidylinositol 3 kinase (PI3K) inhibitors and overcoming drug resistance in various mouse cancer models, including pancreatic, bladder, endometrial, and breast cancer models, as well as acute myeloid leukemia.^145^ KD appears to enhance this effectiveness by decreasing hyperglycemia and reducing insulin secretion, actions correlated with a decrease in mTORC1 signaling within the tumor.^268^

Finally, the combination of serine deprivation and biguanide treatment, such as phenformin and metformin, can lead to metabolic stress in cancer cells. This stress arises from the forced upregulation of glycolysis due to the biguanide-induced reduction in OXPHOS. Under conditions of serine deficiency, this stress may exceed the metabolic flexibility of cancer cells, leading to their potential death and, consequently, enhanced anticancer effects.^269^

In summary, these findings underscore the potential of dietary interventions to modulate the therapeutic landscape of cancer treatment, enhancing the effectiveness of drugs and potentially overcoming resistance mechanisms. However, it should be viewed with cautious optimism. The biological plausibility of diet modifying treatment efficacy and resistance is compelling; however, the translation of this concept into clinical practice requires rigorous validation. It is critical to remain grounded in evidence-based medicine, recognizing that dietary strategies are adjuncts, not replacements, for established therapeutic regimens. Further exploration and clinical validation are necessary to fully understand these interactions and to integrate dietary strategies into standard cancer care effectively and safely.

## Diet changes the gut microbiome in conjunction with antitumor effects and cancer treatment

The gut microbiome encompasses the genetic makeup of all species within the gut, such as bacteria, viruses, yeasts, protozoans, fungi, and archaea, and can be affected by a range of internal and external factors.^270^ The gut microbiota plays a significant role in influencing the health and disease status of the host. The constituents of the gut microbiome and their interactions with the host immune system can impact the development of tumors and carcinogenesis.^271^ Various dietary patterns have been found to significantly influence the composition and functionality of the gut microbiome.^272,273^ It is through these changes in the gut microbiome that dietary patterns can indirectly influence the outcomes of cancer patients.^274^

In recent early studies, several interventional strategies, ranging from dietary interventions to fecal microbiome transplant (FMT) and prebiotic, probiotic and antibiotic treatments, have shown promise in altering the composition or functional capacity of the gut microbiome.^275^ Two prospective cohort studies have suggested that diet-related inflammation can alter the gut microbiome, leading to the development of CRC by suppressing adaptive antitumor immune responses.^276,277^ Other prospective cohort studies have revealed the associations between prudent diets (rich in whole grains and dietary fiber) and Western diets (rich in red and processed meat, refined grains, and desserts) with CRC risk and indicated that the effect of these diets may differ based on the presence of *Fusobacterium nucleatum* in tumor tissue.^278,279^ Specifically, these studies showed that, compared with a Western diet, adhering to a long-term prudent diet is associated with a reduced risk of *F. nucleatum*-positive CRC; however, it does not appear to mitigate the risk of *F. nucleatum*-negative CRC.^278^ A recent study investigated the impact of the gut microbiota and dietary patterns on the response to ICIs in patients with melanoma. The present study revealed that patients with microbiomes dominated by the *Ruminococcaceae* family had greater response rates than did those with microbiomes dominated by the *Bacteroidaceae* family. Furthermore, another finding revealed that a poor response was associated with decreased intake of fiber and omega-3 fatty acids.^280^ These results suggest that dietary interventions may be promising for improving cancer treatment outcomes.

Accumulating data suggest that alterations in the gut microbiome primarily contribute to the progression, prognosis, and treatment of cancer, primarily through interactions with the immune system. Metabolites produced by the microbiota play important roles in modulating antitumor immunity.^281,282^ Microbiota-derived metabolites have been demonstrated to influence the efficacy of tumor immunotherapy. Short-chain fatty acids (SCFAs) are produced primarily by the fermentation of nondigestible carbohydrates, such as dietary fiber, by the microbiota. The main SCFAs include acetate, propionate, and butyrate.^283,284^ The gut microbiota, which is mediated by SCFAs, can potentiate the antitumor activity of CD8^+^ T cells, thereby influencing the efficacy of tumor immunotherapy both in vitro and in vivo.^285^ Metabolic and epigenetic reprogramming enables pentanoate and butyrate to enhance the effectiveness of cancer immunotherapy by boosting the antitumor activity of antigen-specific cytotoxic T lymphocytes and ROR1-targeting chimeric antigen receptor (CAR)-T cells.^286^ Inosine is another important metabolite produced by the microbiome and is closely associated with immunotherapy. Intestinal *Bifidobacterium pseudolongum* promoted Th1 cell transcriptional differentiation and antitumor activity to increase the efficacy of immunotherapy, mainly through the action of inosine.^287^ Inosine is instrumental in enhancing antitumor therapy by serving as a carbon source for CD8^+^ T cells in glucose-restricted microenvironments, facilitating their growth and optimal functioning.^288^ Moreover, engineered bacteria can modify the concentration of metabolites in the microenvironment, thereby altering the composition of the TME. For instance, the genetically engineered probiotic strain *Escherichia coli Nissle 1917* colonizes tumor sites and continuously converts ammonia metabolites into L-arginine. When injected into the tumor, this strain has been shown to increase the concentration of L-arginine within the microenvironment, leading to increased infiltration of tumor-infiltrating T cells, sustained effector T-cell functions, increased tumor-specific T-cell memory formation, and enhanced efficacy of PD-L1-blocking antibodies.^289^

**Fig. 3:**
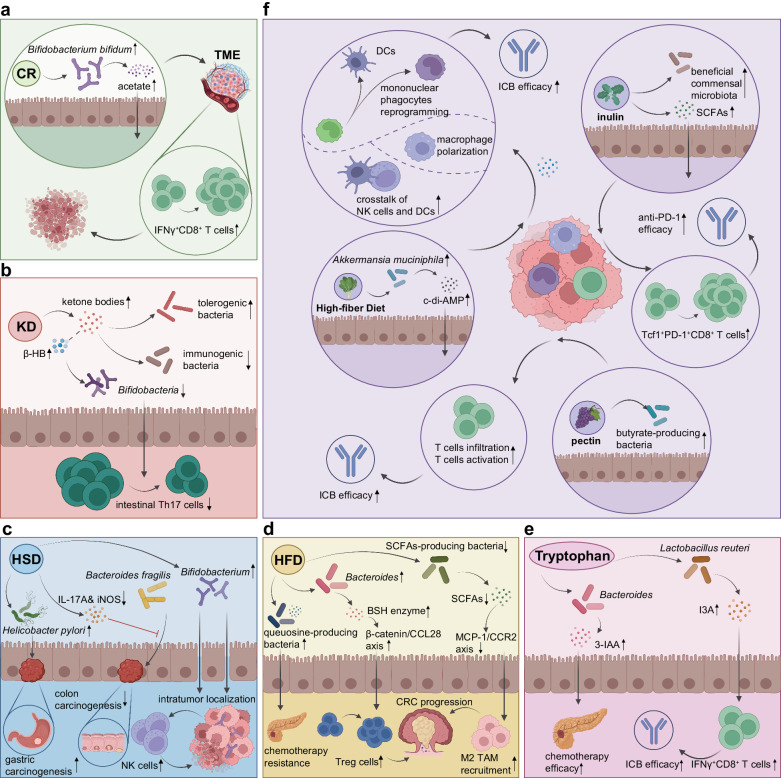
Mechanisms by which diet modulates antitumor effects and cancer treatment via modulation of the gut microbiome. **a** Calorie restriction (CR) elevates IFNγ^+^CD8^+^ T cells in the tumor microenvironment (TME) by enriching *Bifidobacterium bifidum* and increasing acetate levels. **b** Ketogenic diet (KD) induces a shift from tolerogenic (*Lactobacilli* spp., *Clostridium asparagiforme*) toward immunogenic bacteria (such as *Akkermansia muciniphila*) driven by host production of ketone bodies, of which β-HB selectively inhibits the growth of bifidobacteria, resulting in KD-associated decreases in intestinal Th17 cell levels. **c** High-salt diet (HSD) increases the abundance of *Bifidobacterium* and leads to intratumoral localization of *Bifidobacterium*, further enhancing NK cell functions and tumor regression. HSD decreases the expression of IL-17A and iNOS and inhibits inflammation, which reduces enterotoxigenic *Bacteroides fragilis* (ETBF)-promoted colon carcinogenesis. HSD exacerbates *Helicobacter pylori* infection and promotes gastric carcinogenesis. **d** High-fat diet (HFD), through augmentation of queuosine-producing gut bacteria, can incite chemotherapy resistance in pancreatic cancer patients. HFD reduces SCFA-producing bacteria and SCFA production, leading to decreased levels of short-chain fatty acids (SCFAs) that activate the MCP-1/CCR2 axis, which promotes M2 TAM recruitment and polarization, ultimately contributing to colorectal cancer (CRC) progression. High bile salt hydrolase (BSH) enzyme activity in an HFD mouse model activates the β-catenin/CCL28 axis, further inducing immunosuppressive Tregs and accelerating CRC progression. **e** Dietary intake rich in tryptophan stimulates certain *Bacteroides* to produce the metabolite indole-3-acetic acid (3-IAA). Increased levels of 3-IAA enhance the efficacy of chemotherapy treatment. Dietary intake rich in tryptophan, through the action of the probiotic *Lactobacillus reuteri* (Lr), leads to the production of the metabolite indole-3-aldehyde (I3A). This metabolite promotes the production of IFNγ from CD8^+^ T cells, thereby enhancing antitumor immunity and the efficacy of immune checkpoint inhibitors (ICIs). **f** High-fiber diet enriches *Akkermansia muciniphila* which produces the microbiota-derived STING agonist c-di-AMP, inducing type I interferon (IFN-I) production by intratumoural monocytes, resulting in various TME modulation pathways, including reprogramming of mononuclear phagocytes into immunostimulatory monocytes and DCs, promoting macrophage polarization toward an antitumor phenotype and stimulating crosstalk between NK cells and DCs, further enhancing the therapeutic effect of immunotherapy. Dietary fiber inulin can enhance the effectiveness of anti-PD-1 therapy by increasing the abundance of beneficial commensal microbes (e.g., *Akkermansia*, *Lactobacillus* and *Roseburia*) and SCFAs, further increasing the number of stem-like T-cell factor-1 (Tcf1)^+^PD-1^+^CD8^+^ T cells numbers. Dietary fiber pectin can improve the effectiveness of anti-PD-1 therapy by increasing the abundance of butyrate-producing bacteria, further promoting T-cell infiltration and activation in the TME. This figure was created with BioRender.com

Recent research has highlighted the role of the gut microbiota in the antitumor effects of dietary intervention (Fig. 3). Specifically, enrichment of *Bifidobacterium bifidum* after CR increases acetate levels, which in turn elevates IFNγ^+^CD8^+^ T cells in the TME. In contrast, the antitumor effect of IF was not mediated by the gut microbiome, as it was not abrogated after the microbiota was depleted.^290^ Similarly, recent studies have revealed that KD significantly influences the gut microbiota, inducing a shift from a population dominated by tolerogenic bacteria (*Lactobacilli* spp., *Clostridium asparagiforme*) toward a population dominated by an increase in immunogenic bacteria (such as *Akkermansia muciniphila*).^237^ It has been reported that a shift in the gut microbiota is partially attributable to the host’s production of ketone bodies due to the intake of a KD. Among these ketone bodies, β-HB selectively suppresses the proliferation of *Bifidobacterium*. This suppression subsequently leads to a reduction in intestinal Th17 immune cells.^291^ Dietary methionine/cystine restriction has been shown to alter the gut microbiota and potentially contribute to immune system alterations. Specifically, this type of diet restriction promoted a significant decrease in the relative abundance of multiple *Ruminococcaceae* and *Prevotellaceae* families while increasing the presence of members of the *Lactobacillaceae* family.^163^ Consumption of an HSD promotes an increase in the abundance of *Bifidobacterium*, which, due to enhanced gut permeability, infiltrates tumors, subsequently augmenting the functionality of NK cells and ultimately contributing to tumor regression. These results suggest that HSD intake modulates the gut microbiome, which may stimulate NK cell-dependent tumor immunity, thereby providing potential implications for the development of novel therapeutic interventions.^183^ The intake of HSD has also been shown to inhibit enterotoxigenic *Bacteroides fragilis* (ETBF)-promoted colon carcinogenesis by decreasing the expression of IL-17A and iNOS, thereby inhibiting inflammation.^292^ However, intake of an HSD can exacerbate *Helicobacter pylori* infection, contributing to gastric carcinogenesis.^293^ In a mouse model of Barrett’s esophagus, feeding an HFD was observed to promote dysplasia and carcinogenesis by modulating the esophageal microenvironment and gut microbiome, thereby inducing inflammation and promoting stem cell proliferation.^294^ The bile salt hydrolase (BSH) enzyme expressed by Bacteroides was also found to play a crucial role in CRC progression in overweight patients and in model mice with HFD-induced CRC. High BSH activity activates the β-catenin/CCL28 axis, resulting in an increase in immunosuppressive Tregs and accelerated CRC progression.^295^ Moreover, HFD feeding can reduce the level of SCFA-producing bacteria and the rate of SCFA production, leading to decreased levels of SCFAs that can activate the MCP-1/CCR2 axis. This effect promotes M2 TAM recruitment and polarization, ultimately contributing to CRC progression.^296^

Studies suggest that the gut microbiota plays a crucial role in modulating the therapeutic response to immunotherapy.^297,298^ In fact, specific gut microbial signatures have been shown to differentiate responders from nonresponders across various epithelial tumor types in cohorts treated with ICB.^299^ Considering the profound impact of the gut microbiota on the immune system, research investigating the modulation of the gut microbiota via dietary interventions to optimize cancer treatment efficacy has been predominantly centered around immunotherapy. A high-fiber dietary intervention has been associated with significantly prolonged PFS in melanoma patients receiving ICB treatment.^300^ Microbiota-derived STING agonists, specifically c-di-AMP, induce the production of type I interferon (IFN-I) in intratumoral monocytes. This activation results in the transformation of mononuclear phagocytes within the TME into immunostimulatory monocytes and DCs. Additionally, it promotes the polarization of macrophages to antitumor macrophages and stimulates crosstalk between NK cells and DCs. A high-fiber diet can trigger this mechanism by enriching the population of *Akkermansia muciniphila*, which produces c-di-AMP and enhances the therapeutic effect of ICB in melanoma patients.^301^ The presence of *Akkermansia*, a mucin-degrading bacterium, is strongly associated with favorable outcomes in cancer patients.^302^ Moreover, inulin, a polysaccharide dietary fiber, can enhance the effectiveness of anti-PD-1 therapy by increasing the abundance of beneficial commensal microbiota genera (e.g., *Akkermansia*, *Lactobacillus* and *Roseburia*) and SCFAs, further increasing the number of stem-like T-cell factor-1 (Tcf1)^+^PD-1^+^CD8^+^ T cells.^303^ Similarly, oral administration of pectin, another dietary polysaccharide fiber, can largely improve the efficacy of anti-PD-1 mAbs by increasing the number of butyrate-producing bacteria, which is sufficient to promote T-cell infiltration and activation in the TME.^304^

Although research into the antitumor or protumor effects of the intratumor microbiome is still in its early stages, recent studies have started to focus on how the intratumor microbiome can influence the effectiveness of immunotherapy. The colonization of *Bifidobacterium* in the microenvironment, combined with anti-CD47 monoclonal antibody treatment, stimulates the STING signaling pathway and enhances the cross-priming of DCs to upregulate CD8^+^ T cells.^305^ The probiotic *Lactobacillus reuteri* (Lr) within melanoma promotes the local generation of IFNγ by CD8^+^ T cells through the release of its tryptophan breakdown metabolite, indole-3-aldehyde (I3A), thus enhancing ICI efficacy. Dietary intake rich in tryptophan boosts the antitumor immunity induced by Lr and ICI, which is dependent on the CD8^+^ T-cell AhR signaling pathway.^306^

Apart from immunotherapy, recent research has also started to investigate how diet, by influencing the gut microbiota, could affect other forms of cancer treatment. By enriching the gut microbiome with queuosine-producing bacteria, HFD can induce chemotherapy resistance in pancreatic cancer through the upregulation of the oxidative stress protector PRDX1. This resistance can be counteracted by SAM, which is typically produced by bacteria in lean diets, highlighting the influence of diet on chemotherapy effectiveness via gut microbiome adjustments.^307^ Expanding on the theme of diet’s influence on chemotherapy effectiveness in pancreatic cancer, another study revealed that the microbiota-derived tryptophan metabolite indole-3-acetic acid (3-IAA) is enriched in patients responsive to chemotherapy. Through dietary manipulation of tryptophan, an increase in 3-IAA production enhances chemotherapy efficacy by disrupting cancer cell metabolic fitness via increased reactive oxygen species and reduced autophagy.^308^ These findings further emphasize the crucial role of gut microbiota modulation via dietary interventions in cancer treatment outcomes.

Despite the significant progress in this field, the complex relationships among dietary factors, the gut microbiota, and cancer treatment still need to be understood. Each individual’s microbiome is unique, influenced by genetics, diet, environment, and lifestyle, which adds layers of complexity to the task of identifying universally beneficial interventions. Additionally, the development of high-throughput technologies and bioinformatics tools for microbiome analysis will be vital in deciphering these complex interactions. These advancements could enable the identification of biomarkers for microbiome-related treatment responses and the customization of diet-based interventions to enhance the efficacy of cancer therapies. The identification of specific dietary factors and gut microbiota constituents that can enhance the effectiveness of cancer therapies may lead to the development of personalized treatments to improve therapeutic outcomes for cancer patients.

## Implications of dietary intervention for other diseases

Dietary interventions may induce, prevent or delay the progression of various diseases in addition to cancer, which also influence human health and longevity. Healthy dietary patterns that are rich in fiber and beneficial nutrients may reduce the risk of disease, while unhealthy dietary patterns may increase the risk of disease and worsen clinical outcomes.^309^ Here, we summarize preclinical and human studies revealing the implications and mechanisms of various dietary patterns on other diseases in addition to cancer, including neurodegenerative diseases, autoimmune diseases, CVD, and metabolic disorders.

### Neurodegenerative diseases

**Fig. 4:**
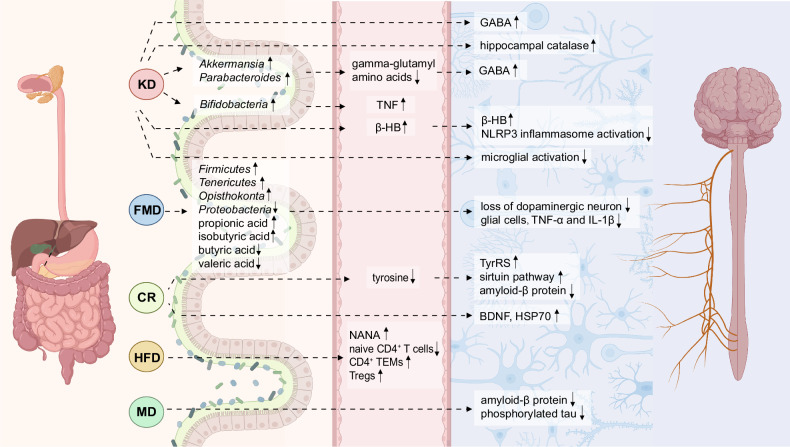
Impact of different diets on neurodegenerative diseases. The ketogenic diet (KD) can enhance inhibitory neurotransmission and anti-inflammatory effects in epilepsy, influence the gut microbiota, and elevate beneficial metabolites. KD is particularly beneficial for treating pediatric drug-resistant epilepsy with elevated specific *Bifidobacteria* and TNF. In Alzheimer’s disease (AD) and Parkinson’s disease (PD), KD could counteract decreased β-HB levels, inhibit the NLRP3 inflammasome, reduce pathology, and alleviate symptoms by inhibiting microglial activation. Fasting mimicking diet (FMD) enhances the gut microbiota composition and metabolites, inhibiting neuroinflammation. This results in the attenuated loss of dopaminergic neurons in the substantia nigra in patients with PD. Caloric restriction (CR) may prevent AD by lowering serum tyrosine levels, reversing the exhaustion of tyrosyl-tRNA synthetase (TyrRS), and upregulating the sirtuin pathway, which attenuates the amyloidogenic processing of amyloid-β protein precursor (APP). Dietary restriction can increase brain-derived neurotrophic factor (BDNF) and chaperone heat-shock protein-70 (HSP70) levels in the striatum and cortex, which are relevant to Huntington’s disease (HD). High-fat diet (HFD) can accelerate recognition-memory impairment in an AD mouse model by increasing blood N-acetylneuraminic acid (NANA) levels, leading to systemic immune exhaustion. Conversely, the Mediterranean diet (MD) may protect against memory decline and mediotemporal atrophy by lowering amyloid-β protein and phosphorylated tau levels, reducing AD risk. This figure was created with BioRender.com

Several neurodegenerative diseases (NDs), such as epilepsy, Alzheimer’s disease (AD), Parkinson’s disease (PD), Huntington’s disease (HD), and amyotrophic lateral sclerosis (ALS), which feature chronic progressive damage to the nervous system, have been proven to be tightly connected with nutrient availability and dietary patterns.^310^ The underlying mechanisms of various dietary interventions mainly include altering neurotransmitters, remodeling, interfering with brain energy metabolism and mitochondrial function, and altering inflammation and oxidative stress. The underlying mechanisms also include altering the composition and balance of the gut microbiome, which further influence the process of neurodegeneration via the gut-brain axis (Fig. 4).

KD has been clinically applied for nearly a century as alternative therapy for childhood intractable epilepsy, but there is sufficient evidence that a modified Atkins diet (MAD) is more tolerable and has a greater probability of causing seizure reduction than a classical KD according to a systematic review.^311–313^ Increased levels of the inhibitory neurotransmitter GABA can be observed in preclinical KD models and patient cerebrospinal fluid (CSF), dampening neuronal excitability.^314–316^ An increase in peroxisome proliferator activated receptor gamma 2 (PPARγ2) and upregulation of hippocampal catalase in KD-fed rats are observed, which may increase anti-inflammatory and antioxidant activity.^317^ In addition, a KD may upregulate potassium channels that are sensitive to ATP opening, reducing the electrical excitability of the brain and increasing the seizure threshold.^318^ The gut microbiota, which includes *Akkermansia*, *Parabacteroides*, and *Bifidobacteria*, also contributes to the neuroprotective effects of KD on epilepsy.^319,320^

Epidemiologic evidence indicates that obesity is an independent risk factor for AD, while HFD is closely associated with an increased risk of obesity.^321^ Recognition-memory impairment in an AD mouse model (5xFAD) can be accelerated by high-fat obesogenic diet by increasing blood levels of the metabolite N-acetylneuraminic acid (NANA), which results in systemic immune exhaustion.^322^ HFD may also enhance neuroinflammation by increasing circulating free fatty acids and cytokines, which may lead to cognitive impairment.^323^ Conversely, healthy dietary interventions, including the Mediterranean diet (MD), CR, and KD, may prevent AD progression.^324–326^ Adhering to MD may act as a protective factor against memory decline and mediotemporal atrophy, as indicated by decreased levels of amyloid-β protein and phosphorylated tau, reducing the risk of AD.^327^ CR may prevent AD by lowering serum tyrosine levels to reverse the exhaustion of tyrosyl-tRNA synthetase (TyrRS) and upregulating the sirtuin pathway, which attenuates the amyloidogenic processing of amyloid-β protein precursor (APP), as confirmed by in vivo and in vitro models.^328,329^ KD may reverse the decreased β-HB levels in red blood cells and the brain parenchyma of AD patients, hence inhibiting NLRP3 inflammasome activation and reducing AD pathology.^330^ In addition, diet can influence AD by modulating the gut microbiome and metabolites. For instance, a Mediterranean-ketogenic diet (MMKD) is associated with improved AD biomarkers in CSF, as indicated by increased *Akkermansia muciniphila* levels, which modulate GABA levels and gut transit time.^331,332^

Gut microenvironmental changes may trigger the development of PD through the gut-brain axis, as determined by the presence of α-synuclein and Lewy bodies in the enteric nervous system and the convincing association between PD and gut inflammation.^333,334^ Research has revealed changes in the gut microbiome in PD patients compared to healthy volunteers, highlighting the potential benefits of dietary interventions in treating PD patients.^335^ High serum sodium is associated with cognitive decline, as observed in the aged population.^336^ However, a recent study denies the association between HSD and neurodegeneration or α-synuclein accumulation in a PLP-hαSyn model, suggesting that the mechanism of HSD needs further exploration.^337^ Adhering to MD is associated with a decreased incidence of PD, the mechanisms of which may include reducing neuroinflammation, similar to AD.^338,339^ KD ameliorates motor and nonmotor symptoms in PD patients by inhibiting microglial activation^340^. FMD promotes a favorable gut microbiota composition and metabolites and inhibits neuroinflammation, consequently attenuating the loss of dopaminergic neurons in the substantia nigra in a PD model.^341^

Other neurodegenerative diseases with lower incidence rates are also relevant to dietary interventions. A clinical trial suggested that increased consumption of dairy products may increase the risk of phenoconversion, resulting in earlier onset of HD.^342^ In addition, high antigliadin antibody titers in patients with HD suggest the potential value of applying gluten-free diet in HD patients.^343^ A dietary restriction regimen retarded the progression of neuropathological, behavioral, and metabolic abnormalities in an HD model, resulting in an extension of life span by increasing brain-derived neurotrophic factor and chaperone heat-shock protein-70 (HSP70) levels in the striatum and cortex, the mechanisms of which still need further explanation.^344^ A cross-sectional baseline analysis revealed that a higher intake of antioxidants and carotenes may result in greater ALS function.^345^ Another meta-analysis revealed that a greater intake of ω-3 PUFAs is associated with a reduced risk of ALS.^346^ Although weight loss has been identified as a negative prognostic factor, high-calorie fatty acid diet provides a significant survival benefit for patients in the subgroup of fast-progressing ALS patients only.^347^

### Autoimmune diseases

**Fig. 5:**
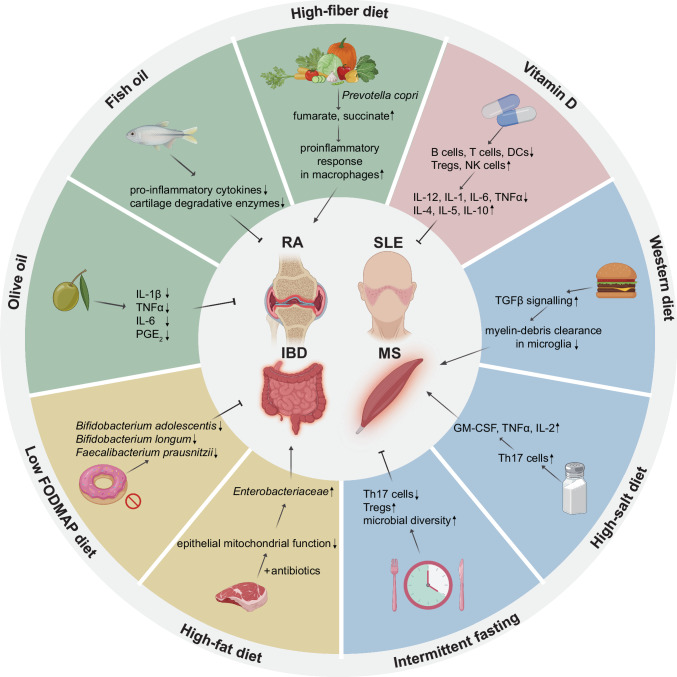
Impact of different diets on autoimmune diseases. Extravirgin olive oil (EVOO) can reduce joint inflammation and degradation in rheumatoid arthritis (RA) due to its phenolic compounds. However, the protective effects of a high-fiber diet can be reversed by *Prevotella copri* colonization, which promotes proinflammatory responses. Fish oil supplementation can suppress proinflammatory cytokines and cartilage degradation, improving RA outcomes. Vitamin D can inhibit the proliferation, differentiation, and function of B and T cells, potentially reducing inflammatory cytokine expression in systemic lupus erythematosus (SLE) patients. A diet low in fermentable oligosaccharides, disaccharides, monosaccharides, and polyols (FODMAPs) can alleviate gut symptoms in quiescent inflammatory bowel disease (IBD) patients, possibly by regulating the immune response through reducing fecal microbial abundance. However, a high-fat diet (HFD) can exacerbate pre-IBD inflammation by impairing epithelial mitochondrial bioenergetics and triggering microbiota disruptions, especially when combined with antibiotics. High salt diet (HSD) can exacerbate autoimmune conditions such as multiple sclerosis (MS) by promoting the induction of pathogenic Th17 cells. Intermittent fasting (IF) can improve MS by reducing the number of IL-17-producing T cells, increasing the number of Tregs in the gut, and enhancing antioxidative microbial metabolic pathways. However, the Western diet can impair myelin-debris clearance in microglia, hindering lesion recovery after demyelination and potentially contributing to MS induction. This figure was created with BioRender.com

Different types of autoimmune diseases, including rheumatoid arthritis (RA), systemic lupus erythematosus (SLE), inflammatory bowel disease (IBD), Hashimoto’s thyroiditis (HT), and multiple sclerosis (MS), can cause distinct clinical features from abnormal activation of the immune system that erroneously attacks healthy host cells and tissues. Impaired gut barrier function, also referred to as a “leaky gut”, which may disrupt the balance between tolerance and immunity to non-self-antigens, is often observed in autoimmune diseases.^348^ This finding suggested a close relationship between diet, the gut, and autoimmune diseases. Dietary interventions may influence the susceptibility, progression and treatment response of these autoimmune diseases through various mechanisms, from adjusting inflammation levels and immune cell composition to adjusting the gut microbiome composition (Fig. 5).

A healthy MD may benefit RA by reducing inflammatory activity and increasing physical function.^349^ Phenolic compounds in extravirgin olive oil (EVOO), an essential component of the MD, can decrease joint edema, cell migration, cartilage degradation and bone erosion by reducing the levels of proinflammatory cytokines and prostaglandin E2 in the joint.^350^ However, the protective effect of high-fiber diet may be reversed if there exists colonization of *Prevotella copri*, which leads to the overproduction of organic acids, including fumarate and succinate, during the digestion of complex fibers and the promotion of proinflammatory responses in macrophages, exacerbating arthritis in an RA model.^351^ In addition, abundant supplementation of fish oil benefits the clinical outcome of RA by suppressing the production of proinflammatory cytokines and cartilage degradative enzymes.^352^ The erythrocyte level of ω-6 PUFAs acts as a biomarker that inverses the risk of RA, and the remission rate of RA increases when ω-3 PUFAs are added to disease-modifying anti-rheumatic drug (DMARD) treatment.^353,354^

Dysbiosis of the gut microbiome can be observed in SLE patients, including a decreased richness and diversity of the gut microbiota and a reduced proportion of Firmicutes/Bacteroides (F/B); the latter may promote lymphocyte activation and Th17 differentiation from naïve CD4^+^ lymphocytes.^355,356^ Blooming of *Ruminococcus (blautia) gnavus* occurs at times of high disease activity and during lupus nephritis, indicating that it is the driver of often remitting-relapsing SLE.^357^ Another analysis showed that *Veillonella dispar* has a positive association with the activity of SLE.^358^ According to a systematic review, nutritional support in the SLE population is focused mainly on interventions involving ω-3 and vitamin D.^359^ The anti-inflammatory effect of ω-3 may contribute to its clinical function, similar to that of RA.^360^ Vitamin D blocks the proliferation, differentiation and function of B cells and T cells, which may attenuate the expression of inflammatory cytokines in patients with SLE.^361^ Inadequate levels of serum vitamin D have been observed in SLE patients, suggesting the importance of supplementing their diet with vitamin D^362^. Dietary patterns other than single nutrients as supplementary treatments for SLE still require further investigation.^363^

Ulcerative colitis (UC) and Crohn’s disease (CD) are the two major clinical phenotypes of IBD. Dietary management and microbiota modulation have been clinically recommended for IBD treatment according to clinical guidelines.^364^ Obesity is a risk factor for IBD, especially for CD.^365^ As a potential trigger of obesity, HFD, together with antibiotics, exacerbates inflammation in pre-IBDs by impairing epithelial mitochondrial bioenergetics and triggering microbiota disruptions in mouse models.^366^ However, IBD increases the risk of malnutrition, which triggers inflammatory responses and subsequently leads to poor clinical outcomes.^367^ Therefore, dietary interventions and nutritional care should be planned according to the precise nutritional assessment and dietary assessment for IBD patients.^368^ Exclusive enteral nutrition (EEN), the first-line therapy in pediatric patients with active CD, can effectively decrease clinical activity and reduce the complications of CD simultaneously, but its benefit in adults still lacks competent evidence.^369^ Similarly, CD exclusion diet (CDED) positively correlates with the clinical remission of pediatric patients with active CD.^370^ In addition, diet low in fermentable oligosaccharides, disaccharides, monosaccharides, and polyols (FODMAPs) can relieve the gut symptoms of patients with quiescent IBD, possibly reducing the fecal abundance of microbes and thereby regulating the immune response of the host.^371^

Dietary interventions may also influence the risk and clinical outcome of other autoimmune diseases. A recent study on HT suggested that low intake of animal foods, mainly meat, has a protective effect on thyroid autoimmunity and potentially has a positive influence on redox balance, which further reduces oxidative stress-related disorders.^372^ Improvement in HT has also been observed in other dietary interventions, including elimination of gluten or lactose, energy restriction, and consumption of Nigella sativa, suggesting the potential benefit of diet as a complementary treatment for HT.^373^ MS is more common in western countries, suggesting diet as a potential risk factor.^374^ Western diet triggers impaired myelin-debris clearance in microglia, thereby impairing lesion recovery after demyelination, which may explain its role in MS induction.^375^ Moreover, an elevated intake of dietary salt can exacerbate autoimmune conditions by promoting the induction of pathogenic Th17 cells, contributing to MS.^376^ Conversely, IF diet ameliorates the clinical course and pathology of MS by reducing the number of IL-17-producing T cells, increasing the number of Tregs in the gut and increasing the richness of gut bacteria, which enhance antioxidative microbial metabolic pathways.^377^ Vitamin D supplementation has been shown to lower the incidence and benefit MS patients with sufficient evidence, and a “Coimbra Protocol” referring to daily doses up to 1000 I.U. vitamin D3 per kg body weight is clinically applied to treat patients with MS.^378,379^

### Cardiovascular diseases (CVD)

According to epidemiological studies, obesity and unhealthy diet are risk factors for CVD. Greater dietary fiber intake from cereal, vegetables and fruits is associated with a lower risk of CVD, suggesting that high-fiber diet is a potential protective factor.^380^ An experimental model fed with diet lack of prebiotic fiber induces hypertension through inducing deficiency of SCFA production and GPR43/109A signaling, suggesting the underlying mechanisms of dietary fiber.^381^ Besides, high-fiber diet and acetate supplementation can lead to changes in the gut microbiota, particularly an increase in *Bacteroides acidifaciens*, which is protective against the development of CVD.^382^ Other healthy dietary patterns, including the Nordic diet, the Dietary Approaches to Stop Hypertension (DASH) diet, the MD, and the vegetarian diet, also have protective effects on CVD risk.^383^ High sodium intake is the leading dietary risk factor for CVD.^384^ High salt load may induce persistent hepatic steatosis and inflammation by inhibiting SIRT3 expression, thereby contributing to cardiovascular damage.^385^ Conversely, a low-sodium diet may dampen the risk of CVD, which is highly recommended by current dietary guidelines.^386^ Amino acids play different roles in the progression of CVD. Diet with high-unsaturated fatty acid composition and less saturated fat might be cardioprotective.^387^ In contrast, higher intake of BCAAs is associated with increased platelet activity and arterial thrombosis formation; therefore, BCAA levels are associated with the risk of CVD.^388^

**Fig. 6:**
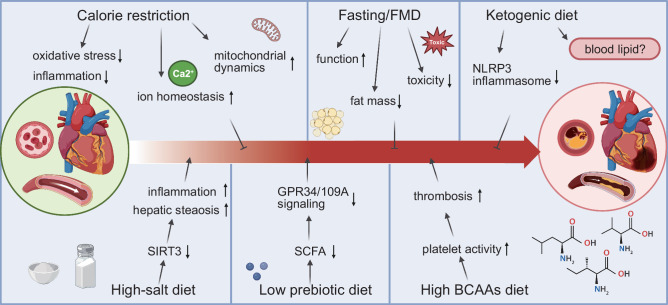
Impact of different diets on cardiovascular diseases. Calorie restriction (CR) can reduce cardiac oxidative stress and inflammation, improve cardiac mitochondrial dynamics and maintain cardiac ion homeostasis, which may be protective against cardiovascular disease (CVD) in obese and/or insulin-resistant models. Fast-mimicking diet (FMD) increases cardiac vascularity and function and resistance to cardiotoxins in a high-fat, high-calorie diet (HFCD) mouse model. Alternate day fasting (ADF) improves cardiovascular markers, for example, reduced fat mass. Ketogenic diet (KD) inhibits the NLRP3 inflammasome and improves the blood lipid profile but may lead to impaired blood lipid profiles in healthy individuals. High-salt diet (HSD) can inhibit SIRT3 expression and induce persistent hepatic steatosis and inflammation, thereby contributing to cardiovascular damage. A diet lacking prebiotic fiber induces hypertension through inducing a deficiency in short-chain fatty acid (SCFA) production and GPR43/109A signaling. High branched-chain amino acid (BCAA) intake is associated with increased platelet activity and arterial thrombosis formation. This figure was created with BioRender.com

Therapeutic implications of diet for CVD treatment have also been a focus of recent studies. CR attenuates hypertension, left ventricular remodeling and diastolic dysfunction in DS/obese rats by reducing cardiac oxidative stress and inflammation.^389^ In addition, a combination of CR and exercise can improve cardiac mitochondrial dynamics, decrease cardiac apoptosis, and maintain cardiac [Ca^2+^]_i_ homeostasis in obese insulin-resistant rats.^390^ CR also helps to maintain the iron homeostasis of cardiomyocytes.^391^ These findings suggest the function of CR in cardiac protection. However, strictly adhering to CR is very difficult for most patients. IF is easier to perform than CR and has similar potential clinical value.^392^ FMD, a 5-day fasting dietary pattern, increases cardiac vascularity and function and resistance to cardiotoxins in a high-fat, high-calorie diet (HFCD) mouse model, thereby postponing the process of cardiac aging.^393^ Alternate day fasting (ADF) improves cardiovascular marker levels, including reduced fat mass, an improved fat-to-lean ratio, and increased β-HB-hydroxybutyrate levels, suggesting its clinical relevance for CVD intervention.^394^ KD has a beneficial effect on the blood lipid profile, the NLRP3 inflammasome, myocardial energy metabolism, and the vascular endothelium, benefiting CVD patients.^395^ However, research on healthy individuals has reported that lipid profiles deteriorate in response to a KD, suggesting that its role in preventing CVD in the normal population needs further inquiry (Fig. 6).^396^

### Metabolic disorders

**Fig. 7:**
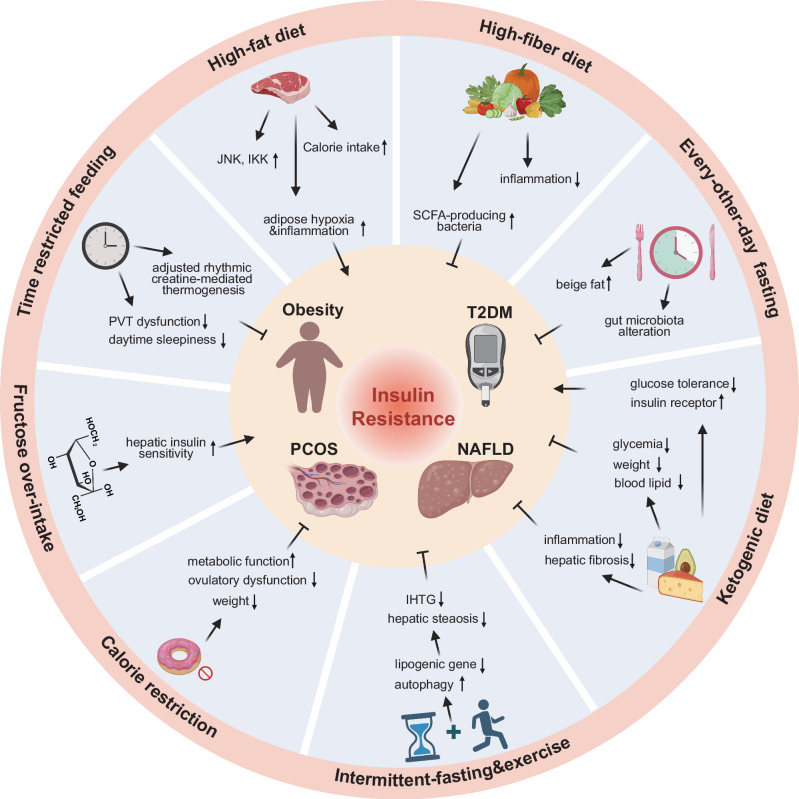
Impact of different diets on metabolic disorders. High-fat diet (HFD) can directly increase caloric intake, induce inflammatory mediators such as JNK and IκB kinase (IKK) to promote hypothalamic inflammation, and contribute to adipose tissue hypoxia and inflammation, which all lead to the development of obesity and/or insulin resistance. Over-intake of fructose can also increase caloric intake and induce obesity by impairing hepatic insulin sensitivity. However, time-restricted feeding (TRF) with equivalent caloric intake from HFD can adjust various signaling pathways and rhythmic creatine-mediated thermogenesis and reverse excessive daytime sleepiness induced by paraventricular thalamic nucleus (PVT) dysfunction, resulting in a protective effect on HFD-induced obesity. High-fiber diet can reduce inflammation and insulin resistance by influencing the gut microbiota and associated molecules, for instance, SCFA-producing bacteria. Every-other-day fasting (EODF) regimen can also shift the gut microbiota composition and stimulate beige fat development within white adipose tissue to inhibit insulin resistance. Ketogenic diet (KD) is clinically beneficial for the glycemic control of type 2 diabetes mellitus (T2DM) and nonalcoholic fatty liver disease (NAFLD). However, in experimental models, KD can decrease sensitivity to peripheral insulin by upregulating insulin receptors. Intermittent fasting (IF) alone or combined with exercise can reduce intrahepatic triglyceride (IHTG) levels and hepatic steatosis in NAFLD patients by downregulating hepatic inflammatory pathways, modifying lipogenic gene expression and inducing autophagy. Calorie restriction (CR) can be effective at reducing weight loss and reversing ovulatory/metabolic dysfunction in polycystic ovarian syndrome (PCOS) patients. This figure was created with BioRender.com

Overnutrition is a driving factor for obesity and related metabolic disorders, mainly including type 2 diabetes mellitus (T2DM), metabolic syndrome, nonalcoholic fatty liver disease (NAFLD), and polycystic ovarian syndrome (PCOS).^397^ In addition, these metabolic disorders have a complicated internal relation, for instance, T2DM and NAFLD are independent factors for each other, and PCOS is closely related to insulin resistance and T2DM.^398,399^ These epidemiological characteristics suggest a high correlation between dietary patterns and multiple metabolic disorders (Fig. 7). Changes in the gut microbiome may also explain the etiology of metabolic disorders by altering the levels of metabolites, such as SCFAs and succinate.^400^

HFD is the standard method to induce obesity in animal models and results from the overconsumption of fat, which directly increases caloric intake. The elevation of inflammatory mediators such as JNK and IκB kinase (IKK) in hypothalamic inflammation may also explain the obesity induced by HFD.^401^ Interestingly, a TRF with equivalent caloric intake from HFD has been shown to have a protective effect on HFD-induced obesity and associated complications by adjusting various signaling pathways and causing rhythmic creatine-mediated thermogenesis, which may further improve nutrient utilization and energy expenditure and reverse excessive daytime sleepiness induced by paraventricular thalamic nucleus (PVT) dysfunction.^402–404^ Adipose tissue hypoxia and inflammation may lead to adipocyte dysfunction and obesity-induced insulin resistance in HFD-fed models, as indicated by increased infiltration of adipose tissue macrophages (ATMs), activation of the NLRP3 inflammasome and increased levels of proinflammatory cytokines.^405–407^ In addition to fat intake, the overintake of fructose may also impair hepatic insulin sensitivity, and several metabolic pathways are independent of increased weight gain and caloric intake.^408^ Within this complex interplay of diet, metabolism, and inflammation, IL-17 has been identified as a key player in metabolic dysregulation associated with HFD, where inhibiting IL-17A production or blocking its receptor can attenuate obesity by enhancing adipose tissue browning and energy dissipation.^409^ Complementarily, IL-17F promote the expression of TGFβ1 in adipocytes, which fosters sympathetic innervation and suggests a novel therapeutic target for obesity that could stimulate thermogenic activity in fat tissue, thereby improving metabolic health and providing a potential treatment strategy for obesity and its related metabolic disorders.^410^

Cohort studies have demonstrated that healthy diets, including the Portfolio diet, DASH diet, and MD, are associated with a decreased risk of T2DM.^411–413^ The promotion of SCFA-producing bacteria induced by dietary fibers observed in T2DM patients suggests the potential value of fiber supplementation in clinical practice.^414^ In addition, increased fiber consumption is associated with decreased insulin resistance, the mechanism of which mainly includes the gut microbiota and associated molecules.^415,416^ IF is an effective strategy for controlling weight and increasing insulin sensitivity in patients with diabetes and can also improve cardiometabolic outcomes.^417,418^ The every-other-day fasting (EODF) regimen selectively stimulates beige fat development within white adipose tissue and shifts the gut microbiota composition in experimental models, explaining the mechanism through which IF ameliorates obesity, insulin resistance, and hepatic steatosis.^419^ KD has therapeutic effects on glycemia, lipid control, and weight reduction in T2DM patients.^420^ However, KD may contribute to decreased sensitivity to peripheral insulin and impaired glucose tolerance by upregulating insulin receptors, as determined by previous studies, which contradicts clinical findings.^421^

NAFLD features hepatic steatosis or adiposity with a potential risk of developing into inflammation, fibrosis, and cancer. MD, as the most recommended dietary pattern for NAFLD, can reduce liver steatosis and improve insulin sensitivity even without weight loss in an insulin-resistant population.^422^ Reduced liver fat may be associated with ameliorated inflammation induced by antioxidants, low glycemic response induced by dietary fiber, and improved hepatic lipid metabolism.^423^ KD is more clinically meaningful for glycemic control in individuals with T2DM and NAFLD than low-calorie diet or high-carbohydrate, low-fat (HCLF) diet.^424,425^ Mechanistically, ketone bodies may modulate inflammation and fibrosis in hepatic cells.^426^ IF alone or combined with exercise is effective at lowering intrahepatic triglyceride (IHTG) levels and reducing hepatic steatosis in patients with NAFLD, possibly by downregulating hepatic inflammatory pathways, modifying lipogenic gene expression and increasing levels of autophagy.^427,428^

PCOS features a series of metabolic irregularities, mainly androgen excess and ovarian dysfunction. A meta-analysis showed that women with PCOS have a lower overall diet quality with higher cholesterol, lower magnesium and lower zinc intake.^429^ Dietary modification with lower caloric intake to achieve weight loss is recommended as a first-line therapy for managing PCOS, and higher supplementary nutrient intake, including vitamin D, chromium and ω-3, may also benefit patients suffering from PCOS.^430^ MD, KD and their combination can all lead to significant improvements in body weight, metabolic function and ovulatory dysfunction in PCOS patients.^431–433^ In addition, IF may be beneficial for treating anovulatory PCOS by reducing body fat and improving menstruation, hyperandrogenemia, insulin resistance and chronic inflammation.^434^ CR may also improve weight and metabolic disorders in patients with PCOS, alone or in combination with supplementation.^435^ However, the exact mechanisms of these dietary interventions remain unclear and need further exploration.

While the potential of dietary interventions to influence systemic diseases of the whole body is supported by various studies, a critical outlook reveals the necessity for more rigorous, long-term clinical trials to validate these findings. It is essential to approach these interventions with caution, considering individual differences and the intricate balance of potential benefits against nutritional deficiencies or other risks.

## Conclusions and perspectives

Our review provides compelling evidence that dietary interventions, including calorie restriction, fasting or FMD, KD, protein restriction diet, HSD, HFD, and high-fiber diet, have substantial potential for modulating metabolism, redirecting disease progression, and enhancing therapeutic responses. These findings highlight the pivotal role of diet, an important environmental factor, in influencing tumor metabolism and the course of various diseases, such as cancer, neurodegenerative diseases, autoimmune diseases, CVD, and metabolic disorders.

Despite compelling evidence, the potential impact of dietary interventions on disease treatment, particularly cancer treatment, is not fully understood.^436^ The latest American Society of Clinical Oncology (ASCO) guidelines suggest that “there is currently insufficient evidence to recommend for or against dietary interventions such as ketogenic or low-carbohydrate diets, low-fat diets, functional foods, or fasting to improve outcomes related to quality of life (QoL), treatment toxicity, or cancer control”.^437^ The intricate relationship between dietary interventions and treatment outcomes can be influenced by numerous factors, such as overall lifestyle habits, health status, specific disease type and its corresponding treatment, degree of dietary alterations, and patient adherence. A comprehensive assessment of these variables is crucial for understanding the precise impact of diet on treatment efficacy.^438,439^

With the recognition of metabolic reprogramming inherent in disease progression, particularly in malignancies, it is becoming essential to explore the value of implementing dietary interventions and translating the evidence into practice. Future research should focus on unraveling the specific molecular mechanisms involved, which will enable the development of more effective, personalized dietary interventions that serve as adjunct therapies in comprehensive disease management.

Building upon the initial observation, it is crucial to interpret and apply these findings with caution due to potential variations and discrepancies. The efficacy of dietary interventions may vary significantly, for instance, depending on the mouse model used.^440^ Each model might have unique metabolic and immune responses that could influence the outcome of dietary interventions. Similarly, the type of cancer cells used to induce tumor formation, whether primary cells derived directly from patient tissues or cultured cell lines, can have profound impacts on the experimental results.^441^ Orthotopic or heterotopic transplantation technique is another significant factor that can influence how tumors respond to dietary interventions. Furthermore, the duration of treatment and the specifics of dietary interventions can substantially influence the results, as short-term interventions might not yield the same results as long-term interventions, and different dietary components could have varying effects on tumor growth and progression.^120^ Therefore, future research in this field should carefully consider the design of animal models and the specifics of dietary interventions to ensure that the findings are robust and translatable to human cancer treatment.

Additionally, clinical trials with larger sample sizes and longer follow-up periods are needed to further validate the efficacy of these strategies and to identify potential side effects and contraindications. It is important for these trials to be designed to represent diverse population groups, including elderly and obese individuals, as these groups may respond differently to dietary modifications. The safety of dietary interventions is another key consideration. While dietary changes generally cause fewer side effects than pharmacological treatments, potential risks should not be overlooked. For instance, severe dietary restrictions may lead to malnutrition or other health complications, particularly in vulnerable population groups. Therefore, in addition to efficacy, these trials should systematically evaluate the safety of dietary interventions, identifying any potential side effects and contraindications.

In conclusion, dietary interventions hold great promise as a novel approach to disease management. However, to realize their full potential, it is essential to continue rigorous scientific investigations into their mechanisms of action, safety profiles, and efficacy in different patient populations. With further research, dietary interventions could become integral components of personalized medicine, providing a new avenue for the prevention and treatment of a myriad of diseases.
